# Pyridoxine requirement of Pacific white shrimp (*Penaeus vannamei*) fed soybean meal based diet

**DOI:** 10.1371/journal.pone.0351680

**Published:** 2026-06-17

**Authors:** Han-Se Kim, Yeonji Lee, Mirasha Hasanthi, Kyeong-Jun Lee

**Affiliations:** 1 Department of Marine Life Sciences, Jeju National University, Jeju, South Korea; 2 Marine Life Research Institute, Jeju National University, Jeju, South Korea; Benha University, EGYPT

## Abstract

This study aimed to determine the dietary pyridoxine requirement of Pacific white shrimp (*Penaeus vannamei*) fed a soybean meal–based diet. Seven experimental diets were prepared with graded pyridoxine supplementation at 0, 25, 50, 75, 100, 125, and 150 mg/kg diet (Con, P25, P50, P75, P100, P125, and P150, respectively). An additional negative control diet (Con^−^) was formulated by adding 0.4% tetracycline hydrochloride to verify microbial synthesis of pyridoxine. Juvenile shrimp (initial weight: 0.38 g) were randomly assigned to eight diets with quadruplicate tanks and fed the diets for 45 days. Growth performance and feed utilization were increased with dietary pyridoxine supplementation up to P75, whereas higher supplementation levels resulted in reduced responses (*P* < 0.001). Hepatopancreas pyridoxine concentration was gradually increased with increment in dietary pyridoxine levels (*P <* 0.001), reaching a plateau from P75 to P150, and remained lower in Con^−^ group than in Con group. Non-specific immune response, including lysozyme, anti-protease and nitroblue tetrazolium activities and antioxidant capacities including superoxide dismutase and glutathione peroxidase were significantly increased by dietary pyridoxine supplementation (*P <* 0.05). Digestive enzyme activities, intestinal morphological indices, and hepatopancreatic F- and B-cell prevalence were also positively influenced by dietary pyridoxine supplementation (*P <* 0.05). Gene expression analysis showed increased transcription of cystathionine-γ-lyase, cystathionine-β-synthase, pyridoxal kinase, crustin, and penaeidine in response to pyridoxine supplementation. Based on a broken-line regression analysis of weight gain, the dietary pyridoxine requirement was estimated to be 69.0 mg/kg diet.

## Introduction

Vitamin B_6_ exists in multiple forms including pyridoxine, pyridoxamine, pyridoxal and their phosphates. Pyridoxine is general term for vitamin B_6_ because it was first isolated and named among B_6_ forms [1]. Each B_6_ form has similar activity *in vivo* after conversion to pyridoxal 5’-phosphate (PLP) as bioactive form but exhibits a different stability due to molecular properties [2]. PLP is a coenzyme involved in amino acid, energy and antioxidant metabolism [3]. In crustaceans, PLP dependent enzymes such as cystathionine-γ-lyase (CGL) and cystathionine-β-synthase (CBS) play key roles in transsulfuration pathway contributing cysteine synthesis and subsequent production of glutathione [4]. In addition, pyridoxal kinase (PK), a key enzyme responsible for the phosphorylation of vitamin B_6_ vitamers, plays a central role in the biosynthesis of PLP, thereby regulating PLP-dependent metabolic processes related to growth, antioxidant defense, and immune function [5]. Previous studies reported that optimal dietary pyridoxine promoted growth performance, digestive enzyme activity and antioxidant capacity in major carp (*Catla catla*) [6], golden pompano (*Trachinotus ovatus*) [7] and yellow catfish (*Pelteobagrus fulvidraco*) [8]. Pyridoxine is known to involve biosynthesis and catabolism of amino acids [5]. Among the different forms of vitamin B_6_, pyridoxine hydrochloride has been recommended as an appropriate additive in extruded feeds for animals due to its high stability under dry and heat-processing conditions [9]. Zehra and Khan [10] reported that spotted snakehead (*Channa punctatus*) exhibited the highest protein efficiency and whole-body protein when their dietary pyridoxine requirement (7.6-10.4 mg/kg diet) was adequately met. Earlier studies reported a close association between dietary pyridoxine supplementation and protein synthetic capacity in Pacific white shrimp (*Penaeus vannamei*) [11,12].

Pyridoxine requirements for the maximum growth have been reported in several invertebrates including black tiger shrimp (*Penaeus monodon*) (72–89 mg/kg diet) [13] and abalone (*Haliotis discus hannai*) (23 mg/kg diet) [14]. Cui et al. [12] suggested that the optimal pyridoxine level is approximately 110 mg/kg diet for *P. vannamei* fed a fish meal (FM) based diet (30% FM in diet). Li et al. [11] reported that the optimal pyridoxine level for *P. vannamei* reared at 3 ppt salinity condition was approximately 152 mg/kg diet for optimal growth. Nutrient requirements of shrimp are known to vary depending on diet formulation, rearing conditions and environmental factors [15]. In response to the limited availability and continuously increasing cost of FM, considerable attention has been directed toward reducing FM levels in shrimp diets [16]. Soybean meal (SM) has been used to replace dietary FM level because of its high digestibility and protein utilization efficiency in *P. vannamei* [17]. A low FM diet containing SM resulted in normal growth and survival of the shrimp [18]. Alvarez et al. [19] reported that *P. vannamei* fed a low FM (13%) with high SM (41%) diet showed growth performance and feed utilization comparable to those of shrimp fed a high FM (29%) diet.

Despite the previous findings on pyridoxine requirement of shrimp species, most available data are derived from FM-based diets. The information on pyridoxine requirement under low FM diet feeding regimes remains very limited. Given the increasing reliance on plant protein ingredients, evaluation of pyridoxine requirement of *P*. *vannamei* is necessary to ensure optimum growth and normal health status when shrimp are fed the plant protein rich diets. Moreover, it was reported that pyridoxine can be synthesized by intestinal microbiota in shrimp, potentially contributing to the host’s vitamin supply [20]. In the present study, a tetracycline-supplemented group (Con−) was therefore employed as a functional control to suppress gut microbiota and delineate the contribution of microbially derived pyridoxine. This approach has been previously applied in *P. vannamei* to discriminate between dietary and microbial vitamin sources, particularly in studies evaluating vitamin B_12_ requirement [21]. We hypothesized that pyridoxine requirement of *P. vannamei* would differ when fed a SM-based diets compared to FM-based formulation, due to differences in nutrient composition and potential changes in metabolic utilization and microbial contribution to vitamin B_6_ synthesis. Therefore, this study was conducted to determine pyridoxine requirement of *P. vannamei* fed a SM-based diet and to assess its effects on the growth performance, feed utilization, digestive enzyme activity, innate immunity, antioxidant capacity, gene expression and gut morphology.

## Materials and methods

### Ethical approval statements

Experimental protocols were conducted in accordance with the guidelines of the Animal Care and Use Committee of Jeju National University (2026−0013), South Korea. All procedures involving animals were approved by Jeju National University.

### Experimental diet

For a basal diet (33% crude protein and 9% crude lipid), 5% of each sardine meal, tuna byproduct meal and squid liver meal were used as the animal protein sources and 46% soybean meal was used as the main plant protein source. Cod liver oil was used as the main dietary lipid source. Two control diets, either containing 0.4% tetracycline hydrochloride (negative control, Con^−^) or not (control, Con), were designed to verify evidence of pyridoxine synthesis by shrimp gut microbiota. The selected tetracycline dose was based on previous studies demonstrating that tetracycline-class antibiotics are safe in *P. vannamei*, with oxytetracycline tolerated up to 0.45% and tetracycline hydrochloride used at 0.3% without adverse effects [21,22]. Six other diets were formulated to contain pyridoxine hydrochloride (purity ≥ 99%, Sigma-Aldrich, St. Louis, MO, USA) levels of 25, 50, 75, 100, 125 and 150 mg/kg diet (designated as P25, P50, P75, P100, P125 and P150, respectively). The graded dietary pyridoxine levels (0–150 mg/kg diet) were established based on previously reported requirement ranges for *P. vannamei*, which have been suggested to be approximately 110–150 mg/kg diet [11,12]. The range of 0–150 mg/kg was designed to encompass both deficient and supraoptimal levels, enabling accurate estimation of the dietary requirement through dose-response analysis. Details of the experimental diet formulation and proximate composition are summarized in Table 1. All dry and fine ingredients were first mixed with cod liver oil and distilled water. And then, the mixture was pelletized using a pelletizer (SP-50, KumKang ENG, Daegu, South Korea) into 2 mm in diameter and dried with a dryer (SI-2400, Shinil General Dryer Co., Ltd, Daegu, South Korea) at 24 °C for 8 h. The dried pellets were kept in a freezer (−20 °C) until used.

**Table 1:** Dietary formulation and proximate composition (%, dry matter) of the experimental diets for the feeding trial of Pacific white shrimp, *Penaeus vannamei.*

Ingredients (%)	Experimental diets							
Con^−^	Con	P25	P50	P75	P100	P125	P150
Fish meal, Sardine^1^	5.00	5.00	5.00	5.00	5.00	5.00	5.00	5.00
Fish meal, Tuna	5.00	5.00	5.00	5.00	5.00	5.00	5.00	5.00
Soybean meal	46.0	46.0	46.0	46.0	46.0	46.0	46.0	46.0
Squid liver meal	5.00	5.00	5.00	5.00	5.00	5.00	5.00	5.00
Wheat flour	9.80	9.80	9.80	9.80	9.80	9.80	9.80	9.80
Cod liver oil^2^	4.00	4.00	4.00	4.00	4.00	4.00	4.00	4.00
Lecithin	1.00	1.00	1.00	1.00	1.00	1.00	1.00	1.00
Cholesterol	0.20	0.20	0.20	0.20	0.20	0.20	0.20	0.20
Mono-calcium phosphate	3.00	3.00	3.00	3.00	3.00	3.00	3.00	3.00
Mineral premix^3^	2.00	2.00	2.00	2.00	2.00	2.00	2.00	2.00
Vitamin premix^4^	1.00	1.00	1.00	1.00	1.00	1.00	1.00	1.00
Pyridoxine hydrochloride (mg/kg)^5^	0.00	0.00	25.0	50.0	75.0	100.0	125.0	150.0
Tetracycline	0.40	0.00	0.00	0.00	0.00	0.00	0.00	0.00
Starch	17.6	18.0	18.0	18.0	18.0	18.0	18.0	18.0
*Proximate composition (%)*								
Crude protein	33.6	33.5	33.3	32.3	33.9	33.7	33.8	33.4
Crude lipid	8.95	8.91	8.54	8.75	8.53	8.95	8.92	8.92
Ash	9.40	9.43	9.44	9.38	9.47	9.54	9.66	9.50
Vitamin B_6_ (mg/kg)	12.7	12.6	24.5	47.6	70.7	93.7	111	135

^1^Orizon S.A., CO., Ltd, Chile.

^2^E-wha oil Industry, Busan, South Korea.

^3^Mineral mixture contained the following amount which were diluted in cellulose (g/kg, mixture): MgSO_4_, 80.2; C_4_H_2_FeO_4_, 12.5; KCl, 130; FeSO_4_. H2O, 20; CuSO_4_. 5H_2_O, 1.25; CoSO_4_, 0.75; Ca (IO_3_)_2_, 0.75; Al (OH)_3_, 0.75; ZnSO_4_. 7H_2_O, 13.75; MnSO_4_, 11.25; CoCl_2_.6H_2_O, 1.

^4^Vitamin mixture contained the following amount which were diluted in cellulose (g/kg, mixture): retinyl acetate, 0.58 g; ergocalciferol, 144.0 g; tocopherol, 20.0 g; menadione, 0.90 g; ascorbic acid, 100.0 g; thiamine hydrochloride, 6.9 g; riboflavin, 18.0 g; nicotinic acid, 18.0 g; calcium pantothenate, 0.9 g; biotin, 7.2g; cyanocobalamin, 0.09g; myo-inositol, 45.0g.

^5^Sigma-Aldrich, St. Louis, MO, USA (purity ≥ 99%)

### Feeding trial

*Pacific white shrimp* juveniles were purchased from a local shrimp hatchery (Tamra shrimp, Jeju, South Korea) and acclimated to the experimental conditions for one week while being fed a commercial diet. For the feeding trial, similar sized shrimp were selected and weighed for mean body weight. Total 640 shrimp (initial body weight: 0.38 ± 0.01 g) were randomly stocked into 32 acrylic tanks (120 L) with 20 shrimp per tank. The tanks were randomly allocated to each dietary treatment in quadruplicates. All the shrimp groups were fed the diets six times (08:00, 10:00, 12:00, 14:00, 16:00 and 18:00) daily for 45 days. As the shrimp weight increased, the daily feeding rate was adjusted from 12 to 4% of their body weight. Residual feed was collected by siphoning 30 min after feeding and used to calculate feed intake by subtracting its dry weight from the total feed offered. The shrimp were bulk-weighed and counted every two weeks to estimate the daily growth rate which was used to adjust daily feeding amounts. During the feeding trial, 70–80% of the rearing water was exchanged with pre-heated sea water every 3 days. The water quality was maintained in suitable for the shrimp rearing under the following conditions: temperature 29.7 ± 1.8 °C, dissolved oxygen 6.32 ± 0.78 mg/L, salinity 30 psu, pH 7.05 ± 0.90 and ammonia 0.08 ± 0.02 mg/L.

### Sample collection

After the feeding trial, shrimp were fasted for 24 h for weighing and sampling. The total number and weight of the shrimp in each tank were measured to calculate weight gain (WG), specific growth rate (SGR), feed conversion ratio (FCR), protein efficiency ratio (PER) and survival. Eight shrimp per tank (32 shrimp per treatment) were randomly taken and anesthetized via ice-cold sea water for sampling. Hemolymph was obtained from the ventral lacuna between bilateral gills (three shrimp per tank) with syringe prefilled with 0.1 mL Alsever’s solution (A3551, Sigma-Aldrich, St. Louis, MO) and transferred in an 2 mL e-tube, then Alsever’s solution was added to achieve a 1:2 ratio of hemolymph to anticoagulant. Anticoagulated hemolymph was centrifuged (800 × *g*, 20 min, 4 °C) and then the plasma was kept in a deep-freezer (−80 °C) for the analyses of innate immunity and antioxidant capacity. Hepatopancreas was collected and frozen in liquid nitrogen immediately for gene expression (three shrimp per tank) and digestive enzyme analyses (three shrimp per tank). For histological examination, the intestine and hepatopancreas (two shrimp per tank) were fixed in Davidson’s solution for 24 h. For whole-body proximate composition analysis, two shrimp from each tank were combined to form a single sample (n = 1 per tank), homogenized, and stored at −20 °C until analysis.

### Diet and whole-body composition analysis

Moisture and ash levels were analyzed based on AOAC methods [23]. Crude protein analysis was performed utilizing a Kjeltec Analyzer (Kjeltec^TM^ 2300, FOSS analytical, Hilleroed, Denmark). Crude lipid was analyzed by Folch et al [24]. Pyridoxine concentration in diets and hepatopancreas was measured with the high-performance liquid chromatography (HPLC) method described in the Ministry of Food and Drug Safety [25]. Briefly, the diet samples (500 mg) were homogenized with 25 mL distilled water. The homogenates were ultrasonicated (40 khz, 30 min, 4 °C), centrifuged (10,000 × *g*, 30 min, 4 °C) and filtered with a filter paper. Then, filtrates were added with distilled water to reach a final volume up to 50 mL. The extract was passed through 0.45 μm syringe filter. Additionally, the hepatopancreas extract was treated with the following ethanol precipitation method to remove remaining protein in the samples. The extract was mixed with ethanol in a ratio of 1: 2. The mixture was kept in a refrigerator (15 min, 4 °C) and then centrifuged (1,700 × *g*, 15 min, 4 °C). Finally, the supernatant was isolated except for the precipitated protein. HPLC (e2695, Waters Alliance, USA) equipped with a reverse T3 column (4.6 mm × 250 mm; 5 μm particle size, Waters Alliance, USA) and a fluorescence detector was performed to detect pyridoxine with 50 mM sodium phosphate solution (pH 2.5) as a mobile phase. Injection volume of sample was 10 μL, column oven temperature was 35 °C, flow rate was 1 mL/min, sample and oven temperature were 25 °C, run time was 40 min and the retention time was 12–13 min.

### Non-specific immune response and antioxidant capacity

Lysozyme activity was measured using a turbidimetric method based on bacterial lysis by enzyme [26]. Briefly, 20 µL of hemolymph was mixed with 200 µL of *Micrococcus lysodeikticus* (M3770, Sigma-Aldrich, St. Louis, MO, USA; 0.75 mg/mL, in 0.1M sodium phosphate buffer pH 6.4) suspension, followed by incubation at 37 °C. The absorbance was recorded at 570 nm at 30 min intervals. Anti-protease activity was assayed by measuring the capacity of trypsin, which was inhibited by anti-protease, to hydrolyze azocasein [27]. Trypsin (T4799, Sigma-Aldrich, St. Louis, MO, USA) solution mixed with 20 µL of hemolymph was incubated at 25 °C for 10 min. Then, 200 µL of phosphate buffer (0.1M, pH 7.0) and 250 µL of 2% azocasein (A2768, Sigma-Aldrich, St. Louis, MO, USA) substrate were added and allowed to incubate at 25 °C for 60 min. The reaction was stopped with 10% trichloroacetic acid (T6399, Sigma-Aldrich, St. Louis, MO, USA). Following centrifugation, the supernatant was reacted with sodium hydroxide (221465, Sigma-Aldrich, St. Louis, MO, USA), and the absorbance was measured at 450 nm. Nitroblue tetrazolium (NBT) activity was measured by quantifying the reactive oxygen species (ROS) production during in the respiratory burst by macrophages [28]. NBT activity was measured by incubating 50 µL of sample with 200 µL of Hank’s balanced salt solution (H6648, Sigma-Aldrich, St. Louis, MO, USA) at 25 °C for 30 min, followed by addition of 100 µL of zymosan and further incubation at 37 °C for 2 h. After incubation, methanol was added, and samples were centrifuged (10,000 × *g*, 15 min, 4 °C). The fixed hemocyte pellet was washed, dried, solubilized in 2 mol/L KOH and dimethyl sulfoxide (D2653, Sigma-Aldrich, St. Louis, MO, USA), and absorbance was measured at 620 nm. Phenoloxidase (PO) activity was measured upon the oxidation level of levodopa occurred by trypsin [29]. For PO activity, 50 µL of hemolymph was combined with 50 µL of trypsin (1 mg/mL in sodium cacodylate buffer) solution and incubated at 25 °C for 10 min. Subsequently, catechol (P0567, Tokyo Chemical Industry, Tokyo, Japan) substrate was added, and the mixture was further incubated at 25 °C for 10 min. The absorbance was then measured at 490 nm. For antioxidant capacity, superoxide dismutase (SOD) activity was analyzed using SOD assay kit (S311-10, Dojindo, Kumamoto, Japan), based on the inhibition of superoxide-mediated reactions. Glutathione peroxidase (GPx) activity was analyzed using GPx assay kits (703102, Cayman Chemical, Ann Arbor, MI, USA), following the oxidation of glutathione coupled with NADPH consumption. All assays were performed according to the manufacturer’s instructions.

### Digestive enzyme analysis

The method described by Schleder et al. [30] was used for the hepatopancreas sample preparation and the analysis of trypsin, chymotrypsin and lipase activities. Hepatopancreas and distilled water were mixed at a ratio of 1: 2 (w/v) and homogenized. The homogenate was centrifuged (15,000 × *g*, 15 min, 4 °C), and the enzymatic extract was obtained, excluding the fat layer. Total protein was analyzed by Bradford [31] method using a Bio-Red protein assay reagent (#5000002, CA, USA) and was used for the calculation of enzymatic activities. Trypsin activity was estimated using n-α-benzoyl-dl-arginine-p-nitroanilide (BAPNA; B4875, Sigma-Aldrich, St. Louis, MO, USA) as a substrate. Briefly, 20 µL of the sample was reacted with 250 µL of BAPNA at 37 °C for 15 min, and absorbance was measured at 410 nm. Chymotrypsin activity was estimated using a 150 µL of succinyl-(ala)2-pro-phe-p-nitroanilide (S7388, Sigma-Aldrich, St. Louis, MO, USA) with 20 µL of the sample at 25 °C. Absorbance was recorded at 410 nm at 5 min intervals. Chymotrypsin and trypsin activities were determined by the amount of released p-nitroanilide by enzyme. Lipase activity was assessed by incubating 20 µL of sample with 45 µL of p-nitrophenol palmitate solution (N2752, Sigma-Aldrich, St. Louis, MO, USA), and 162 µL of 50mM phosphate buffer at 37 °C for 5 min, followed by measuring absorbance at 410 nm. Amylase activity was obtained through calculation of reduced 3,5-dinitrosalicylic acid by maltose which was released by hydrolysis of starch [32]. Briefly, the sample was incubated with a starch solution (33615, Sigma-Alrdish, St. Louis, MO, USA), followed ‌‌by the addition of dinitrosalicylic acid reagent (128848, Sigma-Alrdish, St. Louis, MO, USA). The mixture was then heated at 100 °C for 5 min, diluted with distilled water, and absorbance was measured at 540 nm. Pepsin activity was estimated by calculating the amount of hydrolyzed hemoglobin by enzyme [33]. The reaction mixture consisted of 100 μL of enzyme extract and 500 μL of 2% (w/v) hemoglobin prepared in 0.06 N HCl. The mixture was incubated at 37 °C for 10 min. The reaction was terminated by adding 1 mL of 5% (w/v) trichloroacetic acid, followed by a standing period of 5 min. The mixture was then centrifuged at 12,000 × g for 5 min, and the absorbance of the supernatant was measured at 280 nm.

### Real-time quantitative PCR analysis

For the real-time quantitative PCR (RT-qPCR) analysis, total RNA was extracted from hepatopancreas using TRI-zol^®^ (Sigma-Aldrich) using standard procedures supplied with the commercial kit. The concentration and purity of the RNA extracts were measured using μDrop™ Plate spectrophotometer (Multiskan SkyHigh, Thermo Fisher Scientific, MA, USA) at OD_260_/OD_280_. Complementary DNA (cDNA) was synthesized from total RNA using the cDNA synthesis kit (Takara Bio, Shiga, Japan). Gene expression was performed using RT-qPCR machine (Takara, Shiga, Japan) with β-actin as the reference gene. β-actin served as a reference gene for qPCR normalization, as its expression was not influenced by dietary pyridoxine levels and stably expressed in all dietary treatments. Detailed information on the primers employed for gene amplification is presented in Table 2. Thermal cycling conditions were performed as described in Hasanthi et al. [37]: 1 cycle at 95 °C (10 min), 40 cycles at 95 °C (15 s), 60 °C (30 s) and 72 °C for 30 s; finally 1 cycle at 95 °C (15 s), 60 °C (30 s) and 95 °C (15 s). Relative mRNA expressions of genes were obtained by using a formula [2^−ΔΔCT^; threshold cycles] from Pfaffl [38]. Melt curve analysis was performed after amplification to verify specificity. All reactions showed a single distinct peak indicating no non-specific amplification or primer dimer formation (S1 Fig)

**Table 2:** Primer sequences of target genes used in real-time quantitative PCR.

Gene	Sequence (5′-3′)	Accession number/reference	Amplicon size (bp)
β-actin	F-GAGCAACACGGAGTTCGTTGT R-CATCACCAACTGGGACGACAT	AF300705.2	350
*CBS*	F-AAAATTGTTGGAGTGGACCCCT R-TCGTATCCAATGCCCTCAACTT	[34]	123
*CGL*	F-TTGAGTATGGTCGCTCTGGT R-AGTAAGTGCGTGATGGTGGT	[35]	130
*PK*	F-ACGGAACTTGGCTCAGAGAACGA R-CTTTGCATCTCTGCTCTTGGGCTG	XM027361509.1	146
Penaeidine	F-CACCCTTCGTGAGACCTTTG R- AATATCCCTTTCCCACGTGAC	[36]	162
Crustin	F-CTTGCACACGTGTTCTCCCAAACA R- ACCAAGATACTCGACTGCCCACAA	AY486426.1	142

*CBS*, Cystathionine-β-synthase; *CGL*, Cystathionine-γ-lyase; *PK*, Pyridoxal kinase.

### Histological assessment

For the intestine and hepatopancreas histology, fixed tissues were dehydrated gradually in 70% to 100% ethanol solutions and embedded in paraffin. Samples in paraffin blocks were sliced at 4 µm by a microtome, cleared in xylene to dissolve paraffin and stained with hematoxylin and eosin according to the standard procedures. Then, mounting of the stained tissues was performed by covering with canada balsam (C0320OH1, Daejung Chemicals, Siheung, South Korea) and then examined by using an optical microscope (DM750, Leica, Bensheim, Germany). Wall thickness and epithelium length in the intestine were quantified using image analysis software (Leica Application Suite, version 4.7.1; Leica, Bensheim, Germany). The prevalence of hepatopancreatic cells was evaluated based on the method described by Romano et al. [39]. Twenty tubules were randomly selected from quadruplicate samples per treatment. Then, restzellen cell (R cell), fibrillenzellen cell (F cell) and blasenzellen cell (B cells) were counted by the number of each cell per tubule.

### Statistical analysis

Experimental groups were arranged in a completely randomized design, and statistical analysis was performed using one-way ANOVA in SPSS (version 24.0, IBM Corp., Armonk, NY, USA). Each tank was considered as the experimental unit for statistical analysis, and measurements from multiple shrimp within each tank were averaged to represent one replicate. Prior to analysis, data were tested for normality using the Shapiro-Wilk test and for homogeneity of variances using Levene’s test. Percentage data were arcsine-transformed before anlysis. When assumptions were satisfied, data were analyzed using one-way ANOVA, followed by Tukey’s multiple comparison test to determine significant differences among treatment groups at *P* < 0.05. Orthogonal polynomial contrast analysis was applied to evaluate linear and/or quadratic trends across the graded pyridoxine levels in the Con, P25, P50, P75, P100, P125 and P150 treatments. In addition, the Con^-^ and Con groups were compared separately using an independent T-test. The optimal dietary requirement for pyridoxine was quantified by broken line regression analysis [40]. Data are presented as mean ± SD.

## Results

### Growth performance

**Fig 1:**
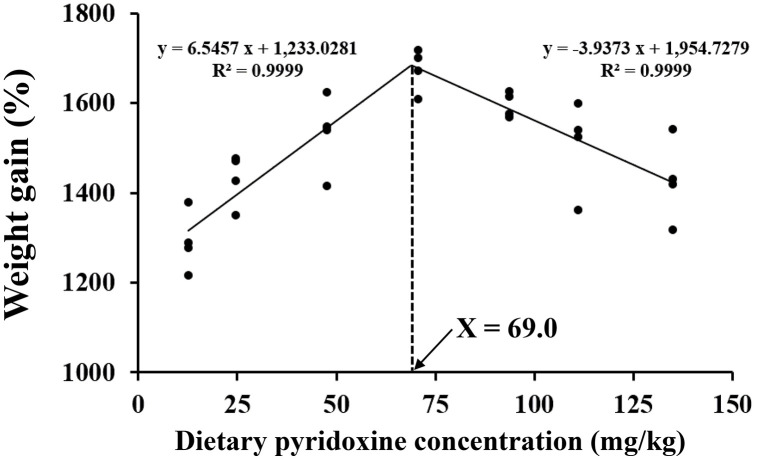
Estimation of optimum dietary pyridoxine requirement using broken-line regression analysis based on the weight gain (%) in Pacific white shrimp (*Penaeus vannamei*). X represents the optimum dietary pyridoxine requirement (69.0 mg/kg) for maximum weight gain (%) in Pacific white shrimp.

The dietary pyridoxine supplementations enhanced the growth performance and feed efficiency (Table 3). FBW, WG, SGR and PER were significantly improved (*P* < 0.001) in P50, P75, P100 and P125 groups than in the Con group. FCR was significantly lower (*P* < 0.001) in P75, P100 and P125 groups than in the Con groups. Growth performance and feed utilization had significant linear and quadratic trends (*P* < 0.05), whereas survival showed no significant trends (*P* ≥ 0.05). The highest growth performance and feed utilization were found in P75 group. Dietary pyridoxine supplementation did not result in significant changes in survival rates among treatments (*P* = 0.985). A broken-line regression analysis showed that the optimal dietary pyridoxine level for the shrimp based on their weight gain was 69.0 mg/kg (Fig. 1). There were no significant differences in growth performance and feed utilization parameters between the Con^−^ and Con groups (*P* ≥ 0.05).

**Table 3:** Growth performance and feed utilization of Pacific white shrimp, *Penaeus vannamei* (initial body weight: 0.38 ± 0.01 g) fed the experimental diets for 45 days.

Diets	FBW^1^(g)	WG^2^ (%)	SGR^3^ (%/day)	FCR^4^	PER^5^	Survival^6^ (%)
Con^−^	5.04 ± 0.27	1225 ± 72	5.74 ± 0.12	1.49 ± 0.06	2.00 ± 0.08	96.3 ± 2.5
Con	5.28 ± 0.25^d^	1291 ± 67^c^	5.85 ± 0.11^c^	1.46 ± 0.05^a^	2.05 ± 0.06^c^	96.3 ± 4.8
P25	5.81 ± 0.22^bcd^	1431 ± 58^bc^	6.06 ± 0.09^bc^	1.42 ± 0.02^ab^	2.12 ± 0.04^bc^	98.8 ± 2.5
P50	6.20 ± 0.33^abc^	1532 ± 86^ab^	6.20 ± 0.12^ab^	1.36 ± 0.05^abc^	2.28 ± 0.08^ab^	97.5 ± 5.0
P75	6.74 ± 0.18^a^	1674 ± 48^a^	6.39 ± 0.06^a^	1.25 ± 0.03^d^	2.36 ± 0.05^a^	98.8 ± 2.5
P100	6.44 ± 0.10^ab^	1596 ± 28^ab^	6.29 ± 0.04^ab^	1.30 ± 0.04 cd	2.29 ± 0.07^ab^	97.5 ± 5.0
P125	6.10 ± 0.39^bc^	1506 ± 102^ab^	6.17 ± 0.14^ab^	1.32 ± 0.08^bcd^	2.25 ± 0.14^ab^	97.5 ± 5.0
P150	5.81 ± 0.35 cd	1427 ± 92^bc^	6.05 ± 0.13^bc^	1.37 ± 0.03^abc^	2.19 ± 0.05^bc^	98.8 ± 2.5
Pr > F^*^						
T-test	0.234	0.228	0.229	0.444	0.387	0.757
ANOVA	<0.001	<0.001	<0.001	<0.001	<0.001	0.985
Linear	0.004	0.004	0.003	<0.001	0.002	0.614
Quadratic	<0.001	<0.001	<0.001	<0.001	<0.000	0.697

Con^–^ and Con diets with and without tetracycline hydrochloride, respectively. P25, P50, P75, P100, P125 and P150, diets supplemented with 25, 50, 75, 100, 125 and 150 mg/kg of pyridoxine. Values are mean of quadruplicate groups and presented as mean ± SD. Values with different superscripts in the same column are significantly different (*P* < 0.05). Orthogonal polynomial contrast was applied for Con, P25, P50, P75, P100, P125 and P150 treatments, and Con^−^ and Con groups were compared using a T-test. ^*^Significance probability is associated with the *F*-statistic.

^1^Final body weight (g/shrimp)

^2^Weight gain (%) = [{Final body weight (g/shrimp) − Initial body weight (g/shrimp)}/Initial body weight (g/shrimp)] × 100

^3^Specific growth rate (%/days) = [{log_e_ Final body weight (g/shrimp) − log_e_ Initial body weight (g/shrimp)}/Days] × 100

^4^Feed conversion ratio = {Dry feed fed (g/tank)}/{Wet body weight gain (g/tank)}

^5^Protein efficiency ratio = {Wet body weight gain (g/tank)}/{Total protein given (g/tank)}

^6^Survival (%) = (Final number of shrimp/Initial number of shrimp) × 100

### Whole-body composition

Hepatopancreas pyridoxine concentration was significantly higher (*P* < 0.001) in all pyridoxine supplemented groups than in the Con group (Table 4). The concentration increased with increasing dietary pyridoxine levels and reached a plateau at 75 mg/kg, with no further significant increases observed in P100, P125 and P150 groups. Whole-body protein was significantly increased (*P* = 0.001) in P75 and P100 groups compared to Con group, whereas crude lipid, ash, and moisture were not significantly (*P* ≥ 0.05) affected by the dietary treatments. Hepatopancreatic pyridoxine concentration in Con^−^ group was significantly lower than that of Con group (*P* = 0.003). Linear and quadratic trends were observed in hepatopancreatic pyridoxine concentration and whole-body protein level with increasing dietary pyridoxine levels (*P* < 0.05).

**Table 4:** Pyridoxine concentration (μg/g) in hepatopancreas and proximate composition (%, wet weight basis) in whole-body of Pacific white shrimp, *Penaeus vannamei* (initial body weight: 0.38 ± 0.01 g) fed the experimental diets for 45 days.

Diets	Pyridoxine (μg/g)	Crude protein	Crude lipid	Ash	Moisture
Con^−^	0.335 ± 0.008	18.2 ± 0.3	1.30 ± 0.13	3.52 ± 0.49	75.9 ± 1.0
Con	0.461 ± 0.004^c^	18.5 ± 0.6^c^	1.33 ± 0.06	3.63 ± 0.24	75.3 ± 0.7
P25	0.666 ± 0.008^b^	18.7 ± 0.4^bc^	1.37 ± 0.07	3.61 ± 0.37	75.9 ± 1.4
P50	0.739 ± 0.038^b^	19.2 ± 0.3^abc^	1.38 ± 0.08	3.59 ± 0.22	75.8 ± 1.1
P75	0.958 ± 0.042^a^	19.8 ± 0.2^a^	1.41 ± 0.05	3.64 ± 0.20	75.3 ± 1.3
P100	1.016 ± 0.024^a^	19.3 ± 0.2^ab^	1.40 ± 0.10	3.62 ± 0.26	75.2 ± 1.1
P125	0.955 ± 0.039^a^	19.0 ± 0.2^abc^	1.39 ± 0.10	3.64 ± 0.43	75.5 ± 0.6
P150	0.945 ± 0.034^a^	18.9 ± 0.2^bc^	1.34 ± 0.09	3.66 ± 0.35	75.4 ± 0.6
Pr > F^*^					
T-test	0.003	0.336	0.696	0.671	0.336
ANOVA	<0.001	0.001	0.728	1.000	0.960
Linear	<0.001	0.045	0.641	0.836	0.732
Quadratic	<0.001	<0.001	0.648	0.843	0.869

Con^–^ and Con diets with and without tetracycline hydrochloride, respectively. P25, P50, P75, P100, P125 and P150, diets supplemented with 25, 50, 75, 100, 125 and 150 mg/kg of pyridoxine. Values are mean of quadruplicate groups and presented as mean ± SD. Values with different superscripts in the same column are significantly different (*P* < 0.05). Orthogonal polynomial contrast was applied for Con, P25, P50, P75, P100, P125 and P150 treatments, and Con^−^ and Con groups were compared using a T-test. ^*^Significance probability is associated with the *F*-statistic.

### Immune and antioxidant responses

Lysozyme activity was significantly increased (*P* = 0.011) in P75, P100 and P125 groups compared to that in the Con group (Table 5). Anti-protease activity was significantly elevated (*P* = 0.008) in P75 group than in Con group. NBT was significantly higher (*P* = 0.001) in P50 and P75 groups than in Con group. PO was not significantly different (*P* = 0.167) among all the groups. Anti-protease activity was significantly higher in Con group than in Con^−^ group (*P* = 0.046). Lysozyme activity exhibited significant linear and quadratic responses to increasing dietary pyridoxine levels (*P* < 0.05), whereas anti-protease, NBT and PO activities showed significant quadratic patterns (*P* < 0.05).

**Table 5:** Hematological innate immunity of Pacific white shrimp, *Penaeus vannamei* (initial body weight: 0.38 ± 0.01 g) fed the experimental diets for 45 days.

Diets	Lysozyme^1^ (U/mL)	Anti-protease^2^ (% inhibition)	NBT^3^ (OD_620_)	PO^4^ (OD_492_)
Con^−^	5.95 ± 0.19	9.8 ± 1.3	3.06 ± 0.32	0.65 ± 0.07
Con	5.94 ± 0.40^b^	11.5 ± 0.5^b^	3.12 ± 0.22^c^	0.66 ± 0.05
P25	6.54 ± 0.10^ab^	12.9 ± 0.9^ab^	3.36 ± 0.12^abc^	0.69 ± 0.06
P50	6.64 ± 0.27^ab^	13.0 ± 0.6^ab^	3.44 ± 0.08^ab^	0.74 ± 0.07
P75	6.91 ± 0.61^a^	14.3 ± 0.6^a^	3.61 ± 0.05^a^	0.80 ± 0.11
P100	6.67 ± 0.17^a^	13.4 ± 1.1^ab^	3.35 ± 0.08^abc^	0.75 ± 0.03
P125	6.71 ± 0.11^a^	13.6 ± 0.6^ab^	3.35 ± 0.09^abc^	0.78 ± 0.13
P150	6.55 ± 0.16^ab^	12.0 ± 1.7^b^	3.31 ± 0.08^bc^	0.72 ± 0.02
Pr > F^*^				
T-test	0.993	0.046	0.789	0.844
ANOVA	0.011	0.008	0.001	0.167
Linear	0.015	0.208	0.132	0.073
Quadratic	0.002	<0.001	<0.001	0.037

Con^–^ and Con diets with and without tetracycline hydrochloride, respectively. P25, P50, P75, P100, P125 and P150, diets supplemented with 25, 50, 75, 100, 125 and 150 mg/kg of pyridoxine. Values are mean of quadruplicate groups and presented as mean ± SD. Values with different superscripts in the same column are significantly different (*P* < 0.05). Orthogonal polynomial contrast was applied for Con, P25, P50, P75, P100, P125 and P150 treatments, and Con^−^ and Con groups were compared using a T-test. * Significance probability is associated with the *F*-statistic.

^1^Lysozyme activity.

^2^Anti-protease activity.

^3^Nitroblue tetrazolium activity.

^4^Phenoloxidase activity.

The hematological antioxidant capacity indices are shown in Table 6. SOD was significantly increased (*P* < 0.001) in all the pyridoxine supplemented groups compared to the Con group. GPx was significantly increased (*P* = 0.006) in P75 group than in the Con group. GPx was significantly higher in Con group than Con^−^ group (*P* = 0.048). Significant linear as well as quadratic responses of SOD and GPx activities to dietary pyridoxine supplementation were detected (P < 0.05).

**Table 6:** Hematological antioxidant capacities of Pacific white shrimp, *Penaeus vannamei* (initial body weight: 0.38 ± 0.01 g) fed the experimental diets for 45 days.

Diets	SOD^1^ (% inhibition)	GPx^2^ (nmol/mL)
Con^−^	69.3 ± 1.0	295 ± 12
Con	69.6 ± 0.8^c^	314 ± 11^b^
P25	76.7 ± 0.5^ab^	329 ± 14^b^
P50	77.4 ± 0.9^ab^	339 ± 10^ab^
P75	78.6 ± 0.4^a^	368 ± 13^a^
P100	76.2 ± 1.0^b^	346 ± 27^ab^
P125	75.8 ± 0.8^b^	340 ± 17^ab^
P150	76.4 ± 1.1^b^	338 ± 12^ab^
Pr > F^*^		
T-test	0.623	0.048
ANOVA	<0.001	0.006
Linear	<0.001	0.028
Quadratic	<0.001	0.002

Con^–^ and Con diets with and without tetracycline hydrochloride, respectively. P25, P50, P75, P100, P125 and P150, diets supplemented with 25, 50, 75, 100, 125 and 150 mg/kg of pyridoxine. Values are mean of quadruplicate groups and presented as mean ± SD. Values with different superscripts in the same column are significantly different (*P* < 0.05). Orthogonal polynomial contrast was applied for Con, P25, P50, P75, P100, P125 and P150 treatments, and Con^−^ and Con groups were compared using a T-test. ^*^Significance probability is associated with the *F*-statistic.

^1^Superoxide dismutase activity

^2^Glutathione peroxidase activity

### Digestive enzyme activity

A significant increase in trypsin activity was observed in the P75 group relative to the Con group (*P* = 0.035), whereas lipase activity showed significant increases in the P50 and P75 groups (*P* = 0.010) (Table 7). Chymotrypsin, pepsin and amylase activities were not significantly affected by the dietary pyridoxine levels (*P* ≥ 0.05). There were no significant differences in digestive enzyme activities between Con and Con^–^ groups (*P* > 0.05). Quadratic trends were observed in trypsin, pepsin, lipase, and amylase activities in response to dietary pyridoxine level (*P* < 0.05).

**Table 7:** Hepatopancreatic digestive enzyme activities of Pacific white shrimp, *Penaeus vannamei* (initial body weight: 0.38 ± 0.01 g) fed the experimental diets for 45 days.

Diets	Trypsin (mU/mg)	Chymotrypsin (mU/mg)	Pepsin (mU/mg)	Lipase (mU/mg)	Amylase (mU/mg)
Con^−^	8.74 ± 0.16	5.07 ± 0.14	8.21 ± 0.38	3.23 ± 0.13	42.4 ± 0.8
Con	8.79 ± 0.51^b^	5.19 ± 0.24	8.20 ± 0.40	3.29 ± 0.06^b^	43.1 ± 0.8
P25	9.66 ± 0.13^ab^	5.30 ± 0.22	8.44 ± 0.43	3.40 ± 0.08^ab^	43.6 ± 1.7
P50	9.60 ± 0.39^ab^	5.36 ± 0.22	8.88 ± 0.34	3.57 ± 0.14^a^	43.8 ± 1.6
P75	9.80 ± 0.39^a^	5.49 ± 0.27	8.86 ± 0.32	3.59 ± 0.16^a^	44.3 ± 1.0
P100	9.32 ± 0.36^ab^	5.44 ± 0.19	8.46 ± 0.23	3.37 ± 0.15^ab^	43.9 ± 0.7
P125	9.15 ± 0.41^ab^	5.44 ± 0.13	8.43 ± 0.45	3.36 ± 0.08^ab^	43.5 ± 0.8
P150	9.20 ± 0.58^ab^	5.42 ± 0.27	8.41 ± 0.34	3.35 ± 0.10^ab^	42.7 ± 0.7
Pr > F^*^					
T-test	0.844	0.435	0.969	0.407	0.251
ANOVA	0.035	0.572	0.131	0.010	0.527
Linear	0.937	0.093	0.846	0.739	0.668
Quadratic	0.005	0.234	0.015	0.001	0.040

Con^–^ and Con diets with and without tetracycline hydrochloride, respectively. P25, P50, P75, P100, P125 and P150, diets supplemented with 25, 50, 75, 100, 125 and 150 mg/kg of pyridoxine. Values are mean of quadruplicate groups and presented as mean ± SD. Values with different superscripts in the same column are significantly different (*P* < 0.05). Orthogonal polynomial contrast was applied for Con, P25, P50, P75, P100, P125 and P150 treatments, and Con^−^ and Con groups were compared using a T-test. ^*^Significance probability is associated with the *F*-statistic.

### Gene expression

The hepatopancreatic *CBS* and *CGL* expressions were significantly upregulated (*P* = 0.013) in P50 and P75 groups than in the Con group (Table 8). *PK* was significantly upregulated (*P* = 0.002) in P75 group than in Con group. Penaeidine and crustin were significantly upregulated (*P* < 0.001) in all the pyridoxine supplemented treatments, with the exception of the P25 group. Gene expression levels did not differ significantly between the Con^–^ and Con groups (*P* ≥ 0.05). The expression of *CGL*, penaeidine and crustin exhibited significant linear trend (*P* < 0.05), while quadratic trends were observed for all analyzed genes.

**Table 8:** Relative mRNA expression in hepatopancreas of Pacific white shrimp (*Penaeus vannamei*) fed the experimental diets for 45 days.

Diets	*CBS* ^1^	*CGL* ^2^	*PK* ^3^	Penaeidine	Crustin
Con^−^	0.89 ± 0.18	0.84 ± 0.13	0.88 ± 0.23	0.81 ± 0.09	0.76 ± 0.28
Con	1.00 ± 0.45^b^	1.00 ± 0.26^b^	1.00 ± 0.21^b^	1.00 ± 0.18^b^	1.00 ± 0.19^d^
P25	1.36 ± 0.38^ab^	1.21 ± 0.15^ab^	1.28 ± 0.19^b^	1.37 ± 0.08^b^	1.49 ± 0.24 cd
P50	1.92 ± 0.37^a^	1.79 ± 0.32^a^	1.33 ± 0.27^ab^	1.50 ± 0.20^a^	1.85 ± 0.31^bc^
P75	1.88 ± 0.37^a^	1.82 ± 0.36^a^	1.75 ± 0.25^a^	1.60 ± 0.12^a^	2.65 ± 0.39^a^
P100	1.57 ± 0.30^ab^	1.63 ± 0.33^ab^	1.36 ± 0.29^ab^	1.72 ± 0.17^a^	2.52 ± 0.34^ab^
P125	1.51 ± 0.26^ab^	1.63 ± 0.55^ab^	1.34 ± 0.10^ab^	1.49 ± 0.14^a^	2.08 ± 0.28^abc^
P150	1.68 ± 0.33^ab^	1.67 ± 0.25 ^ab^	1.24 ± 0.18^b^	1.56 ± 0.27^a^	2.37 ± 0.35^ab^
Pr > F^*^					
T-test	0.632	0.297	0.428	0.090	0.204
ANOVA	0.013	0.013	0.002	<0.001	<0.001
Linear	0.084	0.005	0.099	<0.001	<0.001
Quadratic	0.002	0.011	<0.001	<0.001	<0.001

Con^–^ and Con diets with and without tetracycline hydrochloride, respectively. P25, P50, P75, P100, P125 and P150, diets supplemented with 25, 50, 75, 100, 125 and 150 mg/kg of pyridoxine. Values are mean of quadruplicate groups and presented as mean ± SD. Values with different superscripts in the same column are significantly different (*P* < 0.05). Orthogonal polynomial contrast was applied for Con, P25, P50, P75, P100, P125 and P150 treatments, and Con^−^ and Con groups were compared using a T-test. * Significance probability is associated with the *F*-statistic. Gene expressions were normalized to β-actin and expressed relative to control.

^1^Cystathionine-β-synthase

^2^Cystathionine-γ-lyase

^3^Pyridoxal kinase

### Gut morphology

**Fig 2:**
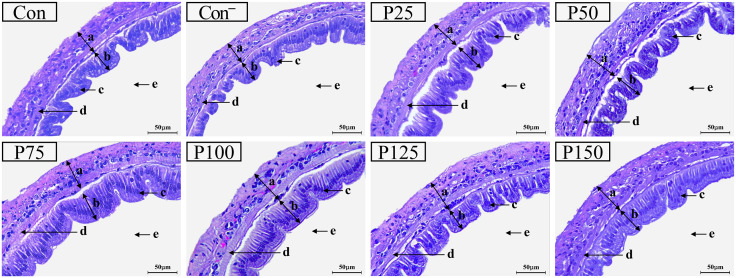
Histological results for intestine of Pacific white shrimp (*Penaeus vannamei*) fed the experimental diets for 45 days. (a) wall thickness, (b) epithelium length, (c) cell nuclei, (d) circular muscle, (e) lumen. (Hematoxylin and eosin stained intestinal tissue section at 40x magnification, scale bar = 50 µm).

**Fig 3:**
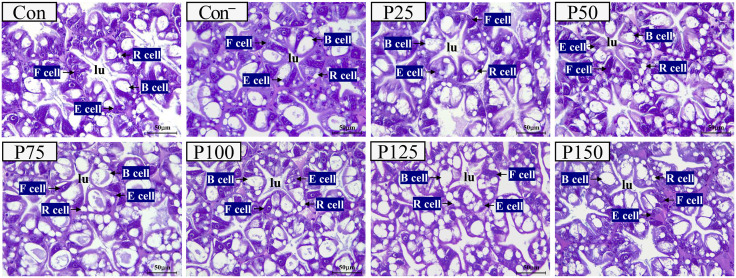
Histological results for hepatopancreas of Pacific white shrimp (*Penaeus vannamei*) fed the experimental diets for 45 days. (B cell) Blasenzellen cell, (E cell) Embryonalzellen cell, (R cell) Restzellen cell, (F cell) Fibrillenzellen cell. (lu) lumen. (Hematoxylin and eosin stained hepatopancreatic tissue section at 40x magnification, scale bar = 50 µm).

Dietary pyridoxine supplementation positively influenced intestinal morphology (Table 9 and Fig. 2). Intestinal wall thickness was markedly increased in the P50, P75, P100, and P125 groups relative to the Con group (P < 0.001). Epithelial length was significantly increased (*P* < 0.001) in P75, P100 and P125 groups compared to the Con group. Both wall thickness and epithelial length were significantly (*P* < 0.05) higher in Con group than in the Con^–^ group. Significant linear and quadratic trends were observed in wall thickness and epithelial length in response to increasing dietary pyridoxine levels. Hepatopancreatic cell prevalence is shown in Table 10 and Fig 3. The abundance of F cell and B cell were significantly higher (*P* < 0.05) in P75 group than in Con group, whereas R cell had no significant difference (*P* = 0.546) among all the groups. The distribution of hepatopancreatic cell types did not differ significantly between Con and Con^–^ groups. Quadratic trends were shown in F cell and B cell abundances.

**Table 9:** The intestinal conditions of Pacific white shrimp, *penaeus vannamei* (initial body weight: 0.38 ± 0.01 g) fed the experimental diets for 45 days.

Diets	Wall thickness (μm)	Epithelium length (μm)
Con^−^	36.9 ± 4.0	31.0 ± 2.7
Con	43.6 ± 3.5^c^	35.1 ± 0.7^d^
P25	46.7 ± 3.5^bc^	37.3 ± 3.9^d^
P50	55.3 ± 3.8^a^	38.0 ± 1.9^d^
P75	58.1 ± 1.6^a^	51.6 ± 1.1^a^
P100	56.2 ± 2.2^a^	47.9 ± 2.0^ab^
P125	53.2 ± 2.5^ab^	44.3 ± 1.5^bc^
P150	50.9 ± 5.3^abc^	40.2 ± 4.6 cd
Pr > F^*^		
T-test	0.045	0.027
ANOVA	<0.001	<0.001
Linear	0.001	<0.001
Quadratic	<0.001	<0.001

Con^–^ and Con diets with and without tetracycline hydrochloride, respectively. P25, P50, P75, P100, P125 and P150, diets supplemented with 25, 50, 75, 100, 125 and 150 mg/kg of pyridoxine. Values are mean of quadruplicate groups and presented as mean ± SD. Values with different superscripts in the same column are significantly different (*P* < 0.05). Orthogonal polynomial contrast was applied for Con, P25, P50, P75, P100, P125 and P150 treatments, and Con^−^ and Con groups were compared using a T-test. ^*^Significance probability is associated with the *F*-statistic.

**Table 10:** The hepatopancreatic cell prevalence of Pacific white shrimp, *Penaeus vannamei* (initial body weight: 0.38 ± 0.01 g) fed the experimental diets for 45 days.

Diets	F cell^1^ (cells/tubule)	B cell^2^ (cells/tubule)	R cell^3^ (cells/tubule)
Con^−^	3.31 ± 0.51	3.18 ± 0.32	9.20 ± 1.34
Con	3.90 ± 0.30^b^	3.66 ± 0.50^b^	9.93 ± 0.85
P25	4.25 ± 0.58^b^	3.93 ± 0.47^b^	10.4 ± 1.4
P50	4.49 ± 0.28^ab^	4.44 ± 0.48^ab^	10.8 ± 1.7
P75	5.13 ± 0.20^a^	5.19 ± 0.42^a^	11.7 ± 1.4
P100	4.59 ± 0.29^ab^	4.76 ± 0.59^ab^	11.3 ± 1.7
P125	4.31 ± 0.34^ab^	4.39 ± 0.52^ab^	11.5 ± 1.0
P150	4.28 ± 0.50^ab^	4.16 ± 0.62^ab^	10.9 ± 1.0
Pr > F^*^			
T-test	0.095	0.150	0.395
ANOVA	0.007	0.009	0.546
Linear	0.190	0.058	0.135
Quadratic	0.001	0.001	0.163

Con^–^ and Con diets with and without tetracycline hydrochloride, respectively. P25, P50, P75, P100, P125 and P150, diets supplemented with 25, 50, 75, 100, 125 and 150 mg/kg of pyridoxine. Values are mean of quadruplicate groups and presented as mean ± SD. Values with different superscripts in the same column are significantly different (*P* < 0.05). Orthogonal polynomial contrast was applied for Con, P25, P50, P75, P100, P125 and P150 treatments, and Con^−^ and Con groups were compared using a T-test. ^*^Significance probability is associated with the *F*-statistic.

^1^Fibrillenzellen cell

^2^Blasenzellen cell

^3^Restzellen cell

## Discussion

The growth-enhancing effects of pyridoxine have been well documented, with evidence indicating that dietary pyridoxine is closely associated with improved growth performance and more efficient nutrient utilization in fish species [6–8]. Similar growth effects have also been observed in shrimp [11–13]. The positive pyridoxine effect on the growth and health conditions of the animals was known to its stimulation through the enzymatic pathways closely related to nutrient utilization [41]. Pyridoxine acts as a coenzyme directly in amino acid metabolism by the synthesis and interconversion of non-essential amino acids [42]. It is also involved in fatty acid metabolism by regulating Δ6-desaturase [43]. Pyridoxine is widely linked to enhanced glycogenolysis [44] and regulates glucose homeostasis [45]. The above physiological and metabolic functions of pyridoxine might have been part of the reasons for the improved growth results in the present study. Pyridoxine requirement depends on growth stage, feeding period and dietary formulations even in the same species. Cui et al. [12] and Li et al. [11] reported that the pyridoxine requirements of *P. vannamei* ranged from 110 mg/kg to 152 mg/kg when fed FM-based high protein diets, whereas the present study showed a lower requirement (69 mg/kg). Results obtained in this study suggest that the dietary pyridoxine requirement of *P. vannamei* fed a SM-based low protein diet can be lower than previous results from FM-based diet [11,12]. These differences may be attributed mainly to the difference in dietary nutrient composition, particularly protein sources, i.e., FM vs SM, including dietary protein levels, shrimp size, and rearing conditions. In particular, the use of plant-based protein sources in the present study likely altered nutrient utilization efficiency, thereby reducing the pyridoxine requirement compared to FM-based diets.

In this study, optimal dietary pyridoxine levels increased whole-body protein level of the shrimp which exhibited a similar pattern to the growth performance. Zehra and Khan [10] reported that an increased PER and whole-body protein were found in spotted snakehead fed a diet containing an optimal level of pyridoxine. Kuruma shrimp (*P. japonicus*) fed an optimal dietary protein and pyridoxine also showed improved growth with increased whole-body protein and PER [46]. One of the reasons for the increased protein utilization by the optimal dietary pyridoxine level in this study may be explained as follows; PLP acts as a precursor for aminotransferases, decarboxylases, racemases and dehydratases in the biosynthesis and degradation of amino acids [47]. These PLP-dependent reactions are stimulated by Schiff base formation between aldehyde group of PLP and α-amino group of amino acids [48]. Pyridoxine seems an important dietary factor to achieve the optimal growth and whole-body protein level by accelerating the protein turnover in the shrimp.

B_6_ vitamers are absorbed in intestine and transported mostly to liver to be converted into their active coenzyme forms [49]. After, they are released into bloodstream by binding to albumin [50] or retained in muscle by combining with glycogen phosphorylase [51]. When B_6_ vitamers exceed in the tissues, they are converted to pyridoxic acid and excreted, or high dose of pyridoxine is immediately excreted without conversion [50]. The hepatopancreas, which mainly metabolizes vitamins, is known as the tissue where a pyridoxine pool occurs in shrimp species [13]. Our results indicated that hepatopancreas pyridoxine concentration reached a peak at dietary level of 75 mg/kg and remained relatively constant in P100, P125 and P150 groups, indicating a saturation of tissue accumulation. Despite this apparent saturation, the shrimp fed higher pyridoxine did not exhibit signs of hypervitaminosis, but showed lower growth, feed efficiency, enzyme activity and gene expression than shrimp fed P75 diet. Similar to the present study, previous studies in some fish species have reported that growth performance increased with increasing dietary pyridoxine levels up to an optimal point, followed by a decline at higher inclusion levels [6,7]. Although the underlying mechanisms responsible for the reduced growth at excessive pyridoxine levels in shrimp remain unclear, the decreased growth observed in the high-dose group (P150) may be associated with metabolic imbalance induced by excess pyridoxine. It has been suggested that high concentrations of pyridoxine can interfere with the conversion to its active form, PLP, potentially leading to reduced metabolic efficiency [52]. Although this mechanism has mainly been demonstrated in mammalian systems, it may also provide a possible explanation for the decreased growth observed at excessive pyridoxine levels in the present study. Overall, these results indicate that dietary pyridoxine up to 75 mg/kg diet could be efficiently utilized in the shrimp to achieve their maximum growth and health conditions.

Cui et al. [12] reported that *P. vannamei* fed a FM–based diet (40% crude protein) supplemented with 120 mg/kg pyridoxine exhibited the highest hepatopancreatic pyridoxine concentration (1.22 μg/g tissue). In the present study, the maximum hepatopancreatic pyridoxine level (1.02 μg/g tissue) was similarly observed at 100 mg/kg dietary pyridoxine in shrimp fed a SM–based low-protein diet (33% crude protein). In both studies, tissue pyridoxine concentration did not increase proportionally with increasing dietary supplementation, suggesting that pyridoxine accumulation is regulated rather than linearly dependent on dietary levels. These comparable hepatopancreatic pyridoxine concentrations observed across studies are consistent with previous findings, indicating that shrimp can maintain vitamin B_6_ homoeostasis despite differences in dietary formulation.

Pyridoxine can be synthesized by plants or bacteria, but not in higher animals [53]. In mammalian gut, pyridoxine can be produced through *de novo* or salvage pathways by some bacterial phyla such as Proteobacteria, Bacteroidetes or Actinobacteria, however, additional pyridoxine intake through the diets is required to meet their requirements [54]. Shrimp cannot synthesize most vitamins themselves. Studies on vitamin biosynthesis by gut microorganisms are very limited in shrimp. It was reported that bacteria proportion related to vitamin synthesis was roughly 12.5% among the gut microbiota of *P. vannamei* [55]. A recent study revealed the existence of *Gammaproteobacteria* and *Epsilonproteobacteria* involved in pyridoxine production in the gut of *P. vannamei* [20]. Thus, we assumed that *P. vannamei* may meet some of pyridoxine requirement through the biosynthesis in the gut microorganisms. In this study, tetracycline which inhibits protein synthesis by attaching transfer RNA to the ribosomal receptor site of microorganisms [56], was used as an antibiotic in Con^−^ diet. The lowered hepatopancreas pyridoxine concentration by Con^−^ group clearly indicates that an antibiotic can change the gut microbiota and subsequently inhibit microbial vitamin synthesis in the shrimp. This is consistent with previous findings showing that oxytetracycline can alter the gut bacterial community structure in shrimp, such as *Penaeus monodon*, indicating that antibiotic exposure can modulate microbial composition without necessarily inducing toxicity [57]. By the use of antibiotic in this study, it seems clear that the pyridoxine quantity synthesized by gut microorganisms is insufficient to meet the shrimp pyridoxine requirement, thus, its dietary supplementation is essential.

In this study, an optimal dietary pyridoxine level was able to achieve a maximum antioxidant capacity in the shrimp. This improvement may be mechanistically linked to the upregulation of CBS and CGL genes observed in this study, which are involved in the transsulfuration pathway leading to glutathione synthesis. Antioxidant enzymes (SOD and GPx) are consistently generated to prevent ROS-induced damage by converting superoxide radicals into non-toxic molecules in the body [58]. Ehrenshaft et al. [59] reported that pyridoxine showed a similar ROS resistance capacity to vitamins C and E which are strong antioxidants. Pyridoxine was reported to have antioxidant capacity for superoxide radicals and lipid peroxidation [60]. Pyridoxine plays an important role as an essential coenzyme in the biosynthesis of glutathione [61], and this function enables glutathione to enhance the activity of antioxidant enzymes such as GPx [5]. The previous studies for fishes discovered that an optimum level of dietary pyridoxine can promote the antioxidant enzyme activities in golden pompano [7], major carp [6], grass carp [62] and yellow catfish [8] which are consistent with the present results. For invertebrate, the increased antioxidant capacity by elevated SOD and CAT activities in *P. vannamei* fed an optimal pyridoxine level [12] explains well the increased antioxidant capacity in the present study. Thus, it seems that pyridoxine can be used as an effective supplement to improve the antioxidant capacity in *P. vannamei.*

In this study, dietary pyridoxine significantly up-regulated the gene expression of PLP-related enzymes such as CBS, CGL and PK in the shrimp. B_6_ vitamers are ultimately converted to PLP *in vivo*, as its molecular structure which enables efficient participation as a cofactor in enzymatic reactions [63]. Therefore, PLP can be absorbed by itself without dephosphorylation and used immediately as a coenzyme *in vivo* [64]. PK and phosphate oxidases are essential enzymes for converting the free forms of B_6_ to the active form, therefore, PLP conversion might have been increased by the increased activities of the enzymes. PLP is an essential coenzyme for CBS and CGL in the process of converting toxic metabolites into non-toxic cysteine [61]. Homocysteine is a cytotoxic substance produced in the metabolism in the body. CBS converts homocysteine to cystathionine, and CGL finally converts cystathionine to cysteine. The above processes are also essential to produce glutathione. Therefore, CBS and CGL can be used as indicators to evaluate the bioavailability of pyridoxine. We hypothesized that PLP conversion and detoxication ability in shrimp could be enhanced by dietary pyridoxine with up-regulating *CBS*, *CGL*, and *PK* gene expression. Also, increased GPx activity in shrimp fed optimal dietary pyridoxine might be due to the promotion of glutathione production by increased expression of *CBS* and *CGL*. This indicates that the enhanced antioxidant enzyme activity is supported at the molecular level by increased expression of PLP-dependent enzymes.

Shrimp are considered to mainly rely on innate immunity including opsonization or phagocytosis to defend pathogens [65]. Innate immunity is the primary defense mechanism in aquatic animals against pathogens and is activated by various humoral and cellular responses through signal transduction pathways [66]. Pyridoxine deficiency may elevate the gene expression of glucocorticoid receptor [67], which may increase the apoptosis by stimulating p38 mitogen-activated protein kinase [68]. Zheng et al. [62] found that pyridoxine deficiency induced decreases in anti-inflammatory cytokines and tight junction-related genes, and increased pathological symptoms, cell apoptosis, and pro-inflammatory cytokines in the gill of grass carp infected with *Flavobacterium*. Pyridoxine deficiency was also reported to downregulate gene expressions of immune related factors such as lysozyme, antimicrobial peptides (AMPs), complements, immunoglobulin M and signaling molecules in grass carp [69]. Cui et al. [12] found an increased lysozyme activity in *P. vannamei* fed optimal dietary pyridoxine level. The present results also indicated that an optimal dietary pyridoxine level had beneficial effects on the innate immunity such as phagocytosis and melanization in *P. vannamei* by promoting phagocytic enzymes or macrophage activity. These immune-enhancing effects of dietary pyridoxine may be mechanistically explained by its role as a precursor of PLP, a key coenzyme involved in one-carbon metabolism and nucleic acid synthesis. PLP-dependent enzymes, such as serine hydroxymethyltransferase, contribute to the generation of one-carbon units required for DNA synthesis, which is essential for immune cell proliferation and activation [5]. In addition, pyridoxine has been reported to regulate immune-related signaling pathways, including target of rapamycin and nuclear factor kappa B, thereby modulating cytokine production and inflammatory responses [62]. These mechanisms may collectively support enhanced immune competence and contribute to the upregulation of immune-related genes, including antimicrobial peptides, as observed in the present study. Our results showed that dietary pyridoxine promoted the gene expression of not only PLP-dependent enzymes but also AMPs in the shrimp. Penaeidins consist of rich-cysteine and rich-proline domains and exhibit the antibacterial activity through growth-inhibition of gram-positive bacteria [70]. Crustins are cysteine-rich antimicrobial peptides that exhibit antibacterial activity against gram-positive and/or gram-negative bacteria, depending on their structural domain composition [71]. To the best of our knowledge, the effect of pyridoxine on the activity of AMPs in invertebrates has not been studied. However, the upregulation of CBS and CGL observed in this study suggests enhanced cysteine biosynthesis via the transsulfuration pathway. Given that cysteine is a key structural component of cysteine-rich AMPs, the increased availability of cysteine may be associated with the upregulation of AMP-related genes. These findings indicate that dietary pyridoxine may enhance innate immunity by up-regulating phagocytosis-related enzymes and AMP-related genes expression through PLP-dependent metabolic pathways.

PLP deficiency causes an exocrine abnormality in pancreas, such as decreased activity of amylase, trypsin and chymotrypsin [72]. Hawk and Hundley [73] confirmed that pyridoxine was required for sufficient gastric acid secretion in rats. Pyridoxine helps to co-catalyze amino acid hydrolysis of α-chymotrypsin by forming pyridoxine-α-chymotrypsin compound which can up-modulate the enzymatic specificity of α-chymotrypsin [74]. Digestion-promoting ability of dietary pyridoxine was proved in Jian carp (*Cyprinus carpio* var. Jian) [75] and yellow catfish [8] by up-regulating digestive enzymes. Kumar et al. [76] found that pyridoxine supplementation increased digestive enzyme activity in milkfish (*Chanos chanos*) exposed to chemical stress up to levels similar or higher than that in unstressed fish. In this study, dietary pyridoxine significantly improved digestive enzyme activity in Pacific white shrimp. It can suggest that pyridoxine is essential to achieve the optimal digestibility and nutrient utilization in the shrimp. This enhancement in digestive enzyme activity may be associated with the improved development of hepatopancreatic cells responsible for enzyme synthesis, as well as enhanced intestinal morphology, including increased epithelium length, which facilitates nutrient absorption [77]. A previous study showed that the optimal level of dietary pyridoxine significantly increased intestinal morphology such as villi length and width and intestinal wall thickness in yellow catfish [8]. Wu et al. [78] figured out that pyridoxine deficiency induced intestinal damage such as oxidative stress, cellular apoptosis and impaired physical barrier function in grass carp by modulating gene expressions related to antioxidant, apoptosis and tight junction. In this study, dietary pyridoxine significantly enhanced intestinal wall thickness and epithelium length of the shrimp. The increased intestinal wall thickness and epithelium length might be contributed to the nutrient absorption and utilization, which could be a reason for the increased growth performance and feed utilization. We assume that higher growth and feed utilization in the shrimp fed an optimal level of pyridoxine could be guaranteed by the increased intestinal physical function and promoted subsequential digestive enzyme activity. These improvements in intestinal structure may complement hepatopancreatic function, thereby enhancing both nutrient digestion and absorption in shrimp.

The hepatopancreas tubules are composed of differentiated cell types with distinct functions; therefore, their histological observations can be used as an indicator of the nutritional status and health condition of shrimp [79]. The main role of each cell type is summarized by Vogt [80]. F cells, composed of abundant rough endoplasmic reticulum and Golgi apparatus, produce digestive enzymes such as proteases, lipases and carbohydrases which are secreted them into the tubular lumen. B cells, secretory cells, are involved in the absorption and synthesis of proteins and the production and secretion of emulsifiers. R cells mainly absorb nutrients and store them in the form of lipids or glycogen and then utilize the stored nutrients when necessary. Embryonic cells, stem cells, can be differentiated into F, B or R cells, and participate in generation and regeneration of hepatocytes through mitosis. This study showed that shrimp fed optimal level of dietary pyridoxine exhibited higher abundance of B and F cells in the hepatopancreas. This finding is consistent with lower digestive enzyme activities observed in the control group fed pyridoxine-deficient diet. These results suggest that pyridoxine may enhance the synthesis and secretion of digestive enzymes and proteins by developing hepatopancreatic cells. Overall, dietary pyridoxine appears to promote hepatopancreatic cellular development, thereby improving the growth performance and feed utilization efficiency in Pacific white shrimp.

However, despite these findings, the applicability of the present results to commercial aquaculture systems may be limited, as multiple environmental and management factors can influence nutrient requirements. In addition, the results are based on a single species and a specific diet formulation, and should be interpreted with caution when applied to other species or production systems. Therefore, further studies under commercial-scale farming conditions are required to validate and refine the dietary pyridoxine requirement of shrimp.

## Conclusion

The present study evaluated the dietary pyridoxine requirement of Pacific white shrimp under low FM, high SM-based diet. The optimal dietary pyridoxine level enhanced growth performance, feed efficiency, protein utilization, whole-body protein, antioxidant capacity, non-specific immunity, digestive enzyme activity, gene expression and gut morphology in the shrimp. Based on growth, the optimal dietary pyridoxine level for *P. vannamei* fed a SM-based diet was estimated to be approximately 69 mg/kg. This requirement was lower than previously reported values derived from FM-based diets, highlighting the influence of diet formulation, particularly protein sources on pyridoxine requirement. Overall, the results indicate that optimal dietary pyridoxine plays a critical role in regulating metabolic, digestive and immune functions in Pacific white shrimp.

## Supporting information

S1 Fig Melt curve analysis of qPCR products. Dissociation curve analysis was performed using the Thermal Cycler Dice Real Time System software following amplification. All reactions exhibited a single distinct peak, confirming amplification specificity and the absence of non-specific products or primer-dimers. The representative curve shown corresponds to the reference gene β-actin.
(DOCX)

## References

[1] György P. Crystalline vitamin B6. J Am Chem Soc. 1938;60(4):983–4. doi: 10.1021/ja01271a505

[2] Rosenberg IH. A history of the isolation and identification of vitamin B(6). Ann Nutr Metab. 2012;61(3):236–8. doi: 10.1159/000343113 23183295

[3] Parra M, Stahl S, Hellmann H. Vitamin B₆ and Its Role in Cell Metabolism and Physiology. Cells. 2018;7(7):84. doi: 10.3390/cells7070084 30037155 PMC6071262

[4] Carballal S, Banerjee R. Overview of cysteine metabolism. Redox Chemistry and Biology of Thiols. Elsevier. 2022. p. 423–50. doi: 10.1016/b978-0-323-90219-9.00016-9

[5] Akhtar MS, Ciji A. Pyridoxine and its biological functions in fish: current knowledge and perspectives in aquaculture. Rev Fish Sci Aquac. 2020;29(2):260–78. doi: 10.1080/23308249.2020.1813081

[6] Khan YM, Khan MA. Optimization of dietary pyridoxine improved growth performance, hematological indices, antioxidant capacity, intestinal enzyme activity, non-specific immune response, and liver pyridoxine concentration of fingerling major carp Catla catla (Hamilton). Aquaculture. 2021;541:736815. doi: 10.1016/j.aquaculture.2021.736815

[7] Huang Q, Lin H, Wang R, Huang Z, Zhou C, Yu W, et al. Effect of dietary vitamin B6 supplementation on growth and intestinal microflora of juvenile golden pompano (Trachinotus ovatus). Aquaculture Research. 2019;50(9):2359–70. doi: 10.1111/are.14117

[8] Li P, Hou D, Zhao H, Huang W, Peng K, Cao J. Dietary pyridoxine effect on growth performance, physiological metabolic parameters, intestinal enzymatic activities and antioxidant status of juvenile yellow catfish (Pelteobagrus fulvidraco). Aquaculture Reports. 2022;24:101153. doi: 10.1016/j.aqrep.2022.101153

[9] EFSA Panel on Additives and Products or Substances used in Animal Feed (FEEDAP). Scientific opinion on the safety and efficacy of vitamin B6 (Pyridoxine hydrochloride) as a feed additive for all animal species. EFSA J. 2011;9(5):2171. 42148410 10.2903/j.efsa.2011.2171 PMC13177355

[10] Zehra S, Khan MA. Dietary pyridoxine requirement of fingerling Channa punctatus (Bloch) based on growth performance, liver pyridoxine concentration, and carcass composition. J Appl Aquac. 2018;30(3):238–55. doi: 10.1080/10454438.2018.1456999

[11] Li E, Yu N, Chen L, Zeng C, Liu L, Qin JG. Dietary Vitamin B6 Requirement of the Pacific White Shrimp, Litopenaeus vannamei, at Low Salinity. Journal of the World Aquaculture Society. 2010;41(5):756–63. doi: 10.1111/j.1749-7345.2010.00417.x

[12] Cui P, Zhou QC, Huang XL, Xia MH. Effect of dietary vitamin B6 on growth, feed utilization, health and non‐specific immune of juvenile Pacific white shrimp, Litopenaeus vannamei. Aquac Nutr. 2016;22(5):1143–51. doi: 10.1111/anu.12365

[13] Shiau S-Y, Wu M-H. Dietary vitamin B6 requirement of grass shrimp, Penaeus monodon. Aquaculture. 2003;225(1–4):397–404. doi: 10.1016/s0044-8486(03)00304-1

[14] MAI K, ZHU W, WU G. PYRIDOXINE REQUIREMENT OF JUVENILE ABALONE, HALIOTIS DISCUS HANNAI INO. Journal of Shellfish Research. 2007;26(3):815–20. doi: 10.2983/0730-8000(2007)26[815:projah]2.0.co;2

[15] NRC. Nutrient requirements of fish and shrimp. Washington, D.C., USA: The National Academies Press. 2011.

[16] Ko D, Lee Y, Kim S, Song S, Kim W, Kim S, et al. Full-fat or defatted black soldier fly (Hermetia illucens) larvae meal as a fish meal replacer in diet for Penaeus vannamei. J Insects Food Feed. 2025;12(1):25–41. doi: 10.1163/23524588-bja10257

[17] Sookying D, Davis DA, Soller Dias da Silva F. A review of the development and application of soybean‐based diets for Pacific white shrimp Litopenaeus vannamei. Aquac Nutr. 2013;19(4):441–8. doi: 10.1111/anu.12050

[18] Dai P, Luan S, Sui J, Cao J, Chen B, Meng X, et al. Insight into genetic potential for growth and survival of the Pacific white shrimp (Litopenaeus vannamei) in the context of low‐protein and low‐fishmeal diet use. Aquac Res. 2022;53(9):3337–45. doi: 10.1111/are.15841

[19] Alvarez JS, Hernández‐Llamas A, Galindo J, Fraga I, García T, Villarreal H. Substitution of fishmeal with soybean meal in practical diets for juvenile white shrimp Litopenaeus schmitti (Pérez‐Farfante & Kensley 1997). Aquac Res. 2007;38(7):689–95. doi: 10.1111/j.1365-2109.2007.01654.x

[20] Valle-Gough RE, Samaniego-Gámez BY, Apodaca-Hernández JE, Chiappa-Carrara FX, Rodríguez-Dorantes M, Arena-Ortiz ML. RNA-Seq Analysis on the Microbiota Associated with the White Shrimp (Litopenaeus vannamei) in Different Stages of Development. Applied Sciences. 2022;12(5):2483. doi: 10.3390/app12052483

[21] Medagoda N, Lee KJ. Vitamin B12 is essential for growth, innate immunity, antioxidant capacity, fatty acid and amino acid metabolism, and ammonia tolerance in Pacific white shrimp, Penaeus vannamei. Marine Biotechnology, 2025;27(5):135. 40920227 10.1007/s10126-025-10512-2

[22] Bray WA, Williams RR, Lightner DV, Lawrence AL. Growth, survival and histological responses of the marine shrimp, Litopenaeus vannamei, to three dosage levels of oxytetracycline. Aquaculture. 2006;258(1–4):97–108. doi: 10.1016/j.aquaculture.2006.04.018

[23] AOAC A of OAC. Official methods of analysis of official analytical chemists international. 18th ed. Arlington, VA, USA: Association of Official Analytical Chemists. 2005.

[24] Folch J, Lees M, Sloane Stanley GH. A simple method for the isolation and purification of total lipides from animal tissues. J Biol Chem. 1957;226(1):497–509. doi: 10.1016/s0021-9258(18)64849-5 13428781

[25] MFDS (Ministry of Food and Drug Safety of the Republic of Korea)., 2023.

[26] Ellis AE. Lysozyme assays. In: Stolen JS, Fletcher TC, Anderson DP, Roberson BS, Van Muiswinkel WB, editors. Techniques in fish immunology. Fair Haven: SOS publications. 1990. p. 101–3.

[27] Ellis AE. Serum antiprotease in fish. In: Stolen JS, Fletcher TC, Anderson DP, Roberson BS, Van Muiswinkel WB, editors. Techniques in fish immunology. Fair Haven: SOS publications. 1990. p. 95–9.

[28] Zhang S, Li J, Wu X, Zhong W, Xian J, Liao S, et al. Effects of different dietary lipid level on the growth, survival and immune-relating genes expression in Pacific white shrimp, Litopenaeus vannamei. Fish Shellfish Immunol. 2013;34(5):1131–8. doi: 10.1016/j.fsi.2013.01.016 23403158

[29] Hernández-López J, Gollas-Galván T, Vargas-Albores F. Activation of the prophenoloxidase system of the brown shrimp Penaeus californiensis Holmes). Comparative Biochemistry and Physiology Part C: Pharmacology, Toxicology and Endocrinology. 1996;113(1):61–6. doi: 10.1016/0742-8413(95)02033-0

[30] Schleder DD, Peruch LGB, Poli MA, Ferreira TH, Silva CP, Andreatta ER, et al. Effect of brown seaweeds on Pacific white shrimp growth performance, gut morphology, digestive enzymes activity and resistance to white spot virus. Aquaculture. 2018;495:359–65. doi: 10.1016/j.aquaculture.2018.06.020

[31] Bradford MM. A rapid and sensitive method for the quantitation of microgram quantities of protein utilizing the principle of protein-dye binding. Anal Biochem. 1976;72:248–54. doi: 10.1016/0003-2697(76)90527-3 942051

[32] Natalia Y. Characterization of digestive enzymes in a carnivorous ornamental fish, the Asian bony tongue Scleropages formosus (Osteoglossidae). Aquaculture. 2004;233(1–4):305–20. doi: 10.1016/j.aquaculture.2003.08.012

[33] Prathiviraj R, Rajeev R, Fernandes H, Rathna K, Lipton AN, Selvin J, et al. A gelatinized lipopeptide diet effectively modulates immune response, disease resistance and gut microbiome in Penaeus vannamei challenged with Vibrio parahaemolyticus. Fish Shellfish Immunol. 2021;112:92–107. doi: 10.1016/j.fsi.2021.02.018 33675990

[34] Rathburn CK, Sharp NJ, Ryan JC, Neely MG, Cook M, Chapman RW, et al. Transcriptomic responses of juvenile Pacific whiteleg shrimp, Litopenaeus vannamei, to hypoxia and hypercapnic hypoxia. Physiol Genomics. 2013;45(17):794–807. doi: 10.1152/physiolgenomics.00043.2013 23821614

[35] Fan L, Wang A, Wu Y. Comparative proteomic identification of the hemocyte response to cold stress in white shrimp, Litopenaeus vannamei. J Proteomics. 2013;80:196–206. doi: 10.1016/j.jprot.2012.12.017 23396037

[36] Shin JH, Shin JB, Eom GH, Lee KJ. Effects of dietary mealworm Tenebrio molitor larvae and black soldier fly Hermetia illucens larvae on Pacific white shrimp Litopenaeus vannamei: innate immune responses, anti-oxidant enzyme activity, disease resistance against Vibrio parahaemolyticus and growth. Korean J Fish Aquatic Science. 2021;54(5):624–33. doi: 10.5657/KFAS.2021.0624

[37] Hasanthi M, Chotikachinda R, Medagoda N, Lee KJ. Exogenous protease supplementation in high-and low-fishmeal diets for Pacific white shrimp (Penaeus vannamei): Comparative effect on growth, immunity, nutrient digestibility and gut health. Animal Nutrition. 2025. doi: 10.1016/j.aninu.2025.04.002 PMC12570180 41170399

[38] Pfaffl MW. A new mathematical model for relative quantification in real-time RT-PCR. Nucleic Acids Res. 2001;29(9):e45. doi: 10.1093/nar/29.9.e45 11328886 PMC55695

[39] Romano N, Koh C-B, Ng W-K. Dietary microencapsulated organic acids blend enhances growth, phosphorus utilization, immune response, hepatopancreatic integrity and resistance against Vibrio harveyi in white shrimp, Litopenaeus vannamei. Aquaculture. 2015;435:228–36. doi: 10.1016/j.aquaculture.2014.09.037

[40] Robbins KR. A method, SAS program, and example for fitting the broken-line to growth data. 4. 1986.

[41] Da Silva VR, Gregory III JF. Vitamin B6. In: Marriott BP, Birt DF, Stallings VA, Yates AA, editors. Present knowledge in nutrition. Academic Press. 2020. p. 225–37.

[42] WILLIAMS MA. Vitamin b6 and amino acids--recent research in animals. Vitam Horm. 1964;22:561–79. doi: 10.1016/s0083-6729(08)60352-0 14284118

[43] Tsuge H, Hotta N, Hayakawa T. Effects of vitamin B-6 on (n-3) polyunsaturated fatty acid metabolism. J Nutr. 2000;130(2S Suppl):333S-334S. doi: 10.1093/jn/130.2.333S 10721899

[44] Okada M, Ishikawa K, Watanabe K. Effect of vitamin B6 deficiency on glycogen metabolism in the skeletal muscle, heart, and liver of rats. J Nutr Sci Vitaminol (Tokyo). 1991;37(4):349–57. doi: 10.3177/jnsv.37.349 1765839

[45] Bowling FG. Pyridoxine supply in human development. Semin Cell Dev Biol. 2011;22(6):611–8. doi: 10.1016/j.semcdb.2011.05.003 21664474

[46] Giri INA, Teshima S, Kanazawa A, Ishikawa M. Effects of dietary pyridoxine and protein levels on growth, vitamin B6 content, and free amino acid profile of juvenile Penaeus japonicus. Aquaculture. 1997;157(3–4):263–75. doi: 10.1016/s0044-8486(97)00157-9

[47] Stover PJ, Field MS. Vitamin B-6. Adv Nutr. 2015;6(1):132–3. doi: 10.3945/an.113.005207 25593152 PMC4288272

[48] Casasnovas R, Adrover M, Ortega-Castro J, Frau J, Donoso J, Muñoz F. C-H activation in pyridoxal-5’-phosphate Schiff bases: the role of the imine nitrogen. A combined experimental and computational study. J Phys Chem B. 2012;116(35):10665–75. doi: 10.1021/jp303678n 22845654

[49] Mehansho H, Buss DD, Hamm MW, Henderson LM. Transport and metabolism of pyridoxine in rat liver. Biochim Biophys Acta. 1980;631(1):112–23. doi: 10.1016/0304-4165(80)90059-8 7397240

[50] Bender DA. Vitamin B6: Physiology. Encyclopedia of Human Nutrition. 2013;340–50. doi: 10.1016/B978-0-12-375083-9.00275-0

[51] Black AL, Guirard BM, Snell EE. Increased muscle phosphorylase in rats fed high levels of vitamin B6. J Nutr. 1977;107(11):1962–8. doi: 10.1093/jn/107.11.1962 908952

[52] Vrolijk MF, Opperhuizen A, Jansen EHJM, Hageman GJ, Bast A, Haenen GRMM. The vitamin B6 paradox: Supplementation with high concentrations of pyridoxine leads to decreased vitamin B6 function. Toxicol In Vitro. 2017;44:206–12. doi: 10.1016/j.tiv.2017.07.009 28716455

[53] Fitzpatrick TB. Vitamin B6 in plants: more than meets the eye. Academic Press. 2011. doi: 10.1016/B978-0-12-385853-5.00006-4

[54] Yoshii K, Hosomi K, Sawane K, Kunisawa J. Metabolism of Dietary and Microbial Vitamin B Family in the Regulation of Host Immunity. Front Nutr. 2019;6:48. doi: 10.3389/fnut.2019.00048 31058161 PMC6478888

[55] Alvarez-Ruiz SAP, Luna-González A, Escamilla-Montes R, Fierro-Coronado A, Diarte-Plata G, García-Gutiérrez C, et al. Gut bacterial profile associated with healthy and diseased (AHPND) shrimp Penaeus vannamei. Lat Am J Aquat Res. 2022;50(2):197–211.

[56] Chopra I, Roberts M. Tetracycline antibiotics: mode of action, applications, molecular biology, and epidemiology of bacterial resistance. Microbiol Mol Biol Rev. 2001;65(2):232-60; second page, table of contents. doi: 10.1128/MMBR.65.2.232-260.2001 11381101 PMC99026

[57] Halder S, Mandal SC, Kundu GK, Hossain A, Rabbane MG, Rahman A, Haque MIM. Effects of probiotic and antibiotic on gut microbiome and antibiotic resistance genes in black tiger shrimp (Penaeus monodon) in Bangladesh. Aquaculture Reports. 2026;46:103294.

[58] Harris ED. Regulation of antioxidant enzymes. FASEB J. 1992;6(9):2675–83. doi: 10.1096/fasebj.6.9.1612291 1612291

[59] Ehrenshaft M, Bilski P, Li MY, Chignell CF, Daub ME. A highly conserved sequence is a novel gene involved in de novo vitamin B6 biosynthesis. Proc Natl Acad Sci U S A. 1999;96(16):9374–8. doi: 10.1073/pnas.96.16.9374 10430950 PMC17790

[60] Jain SK, Lim G. Pyridoxine and pyridoxamine inhibits superoxide radicals and prevents lipid peroxidation, protein glycosylation, and (Na K)-ATPase activity reduction in high glucose-treated human erythrocytes. Free Radic Biol Med. 2001;30(3):232–7. doi: 10.1016/S0891-5849(00)00462-7 11165869

[61] Depeint F, Bruce WR, Shangari N, Mehta R, O’Brien PJ. Mitochondrial function and toxicity: role of B vitamins on the one-carbon transfer pathways. Chem Biol Interact. 2006;163(1–2):113–32. doi: 10.1016/j.cbi.2006.05.010 16814759

[62] Zheng X, Feng L, Jiang W-D, Wu P, Liu Y, Kuang S-Y, et al. The regulatory effects of pyridoxine deficiency on the grass carp (Ctenopharyngodon idella) gill barriers immunity, apoptosis, antioxidant, and tight junction challenged with Flavobacterium columnar. Fish Shellfish Immunol. 2020;105:209–23. doi: 10.1016/j.fsi.2020.07.036 32707298

[63] Liang J, Han Q, Tan Y, Ding H, Li J. Current Advances on Structure-Function Relationships of Pyridoxal 5’-Phosphate-Dependent Enzymes. Front Mol Biosci. 2019;6:4. doi: 10.3389/fmolb.2019.00004 30891451 PMC6411801

[64] Morita E, Shirakami Y, Mizuno N. Intestinal absorption of pyridoxal 5’-phosphate at physiological levels in rats. J Nutr Sci Vitaminol (Tokyo). 1988;34(6):553–65. doi: 10.3177/jnsv.34.553 3244043

[65] Kulkarni A, Krishnan S, Anand D, Kokkattunivarthil Uthaman S, Otta SK, Karunasagar I, et al. Immune responses and immunoprotection in crustaceans with special reference to shrimp. Reviews in Aquaculture. 2021;13(1):431–59. doi: 10.1111/raq.12482

[66] Borregaard N, Elsbach P, Ganz T, Garred P, Svejgaard A. Innate immunity: from plants to humans. Immunol Today. 2000;21(2):68–70. doi: 10.1016/s0167-5699(99)01570-4 10652463

[67] Allgood VE, Cidlowski JA. Vitamin B6 modulates transcriptional activation by multiple members of the steroid hormone receptor superfamily. J Biol Chem. 1992;267(6):3819–24. doi: 10.1016/s0021-9258(19)50599-3 1310983

[68] Miller AL, Webb MS, Copik AJ, Wang Y, Johnson BH, Kumar R, et al. p38 Mitogen-activated protein kinase (MAPK) is a key mediator in glucocorticoid-induced apoptosis of lymphoid cells: correlation between p38 MAPK activation and site-specific phosphorylation of the human glucocorticoid receptor at serine 211. Mol Endocrinol. 2005;19(6):1569–83. doi: 10.1210/me.2004-0528 15817653

[69] Zheng X, Feng L, Jiang W-D, Wu P, Liu Y, Jiang J, et al. Dietary pyridoxine deficiency reduced growth performance and impaired intestinal immune function associated with TOR and NF-κB signalling of young grass carp (Ctenopharyngodon idella). Fish Shellfish Immunol. 2017;70:682–700. doi: 10.1016/j.fsi.2017.09.055 28951222

[70] Bachère E, Gueguen Y, Gonzalez M, de Lorgeril J, Garnier J, Romestand B. Insights into the anti-microbial defense of marine invertebrates: the penaeid shrimps and the oyster Crassostrea gigas. Immunol Rev. 2004;198:149–68. doi: 10.1111/j.0105-2896.2004.00115.x 15199961

[71] Bin Hafeez A, Jiang X, Bergen PJ, Zhu Y. Antimicrobial Peptides: An Update on Classifications and Databases. Int J Mol Sci. 2021;22(21):11691. doi: 10.3390/ijms222111691 34769122 PMC8583803

[72] Dubick MA, Gretz D, Majumdar AP. Overt vitamin B-6 deficiency affects rat pancreatic digestive enzyme and glutathione reductase activities. J Nutr. 1995;125(1):20–5. doi: 10.1093/jn/125.1.20 7529303

[73] HAWK EA, HUNDLEY JM. Effect of certain B vitamin deficiencies on gastric secretion in the rat. Proc Soc Exp Biol Med. 1951;78(1):318–22. doi: 10.3181/00379727-78-19060 14892006

[74] Previero A, Kraicsovits F, Pugnière M, Coletti-Previero MA. Modulation of α-chymotrypsin specificity induced by pyridoxine ridoxal. Biotechnil Lett. 1981;3:571–6. doi: 10.1007/BF00133436

[75] He W, Zhou XQ, Feng L, Jiang J, Liu Y. Dietary pyridoxine requirement of juvenile Jian carp (Cyprinus carpio var. Jian). Aquaculture Nutrition. 2009;15(4):402–8. doi: 10.1111/j.1365-2095.2008.00604.x

[76] Kumar N, Ambasankar K, Krishnani KK, Gupta SK, Minhas PS. Dietary pyridoxine promotes growth and cellular metabolic plasticity of Chanos chanos fingerlings exposed to endosulfan induced stress. Aquaculture Research. 2017;48(5):2074–87. doi: 10.1111/are.13042

[77] Lee Y, Lee KJ. Vitamin D3 improves growth performance, digestive enzyme activity, antioxidant capacity, lipid metabolism, histomorphology, Ca and P homeostasis and ammonia stress resistance in Pacific white shrimp (Penaeus vannamei). Marine Biotechnology. 2025;27(4):118. 40705125 10.1007/s10126-025-10497-y

[78] Wu P, Zheng X, Zhou X-Q, Jiang W-D, Liu Y, Jiang J, et al. Deficiency of dietary pyridoxine disturbed the intestinal physical barrier function of young grass carp (Ctenopharyngodon idella). Fish Shellfish Immunol. 2018;74:459–73. doi: 10.1016/j.fsi.2018.01.015 29339045

[79] Vogt G, Storch V, Quinitio ET, Pascual FP. Midgut gland as monitor organ for the nutritional value of diets in Penaeus monodon (Decapoda). Aquaculture. 1985;48(1):1–12. doi: 10.1016/0044-8486(85)90047-x

[80] Vogt G. Functional cytology of the hepatopancreas of decapod crustaceans. J Morphol. 2019;280(9):1405–44. doi: 10.1002/jmor.21040 31298794

