# Supplementary material for: Pyridoxine requirement of Pacific white shrimp (Penaeus vannamei) fed soybean meal based diet
Source: PLoS One. 2026 Jun 17;21(6):e0351680. doi: 10.1371/journal.pone.0351680 (PMC13274893; doi:10.1371/journal.pone.0351680)
Supplement: S1 Fig — Dissociation curve analysis was performed using the Thermal Cycler Dice Real Time System software following amplification. All reactions exhibited a single distinct peak, confirming amplification specificity and the absence of non-specific products or primer-dimers. The representative curve shown corresponds to the reference gene β-actin. (DOCX) [file pone.0351680.s001.docx]

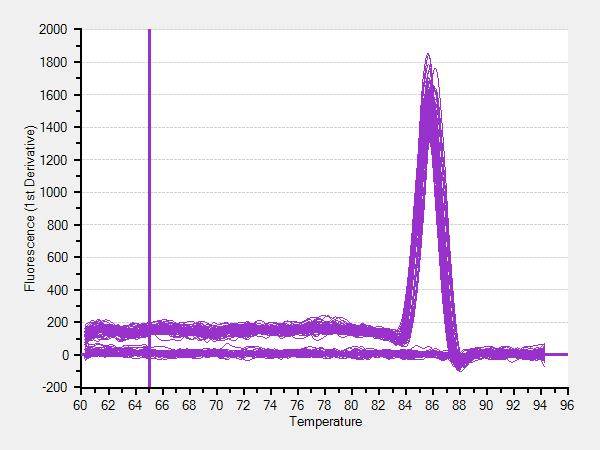


Fig. S1. Melt curve analysis of qPCR products. Dissociation curve analysis was performed using the Thermal Cycler Dice Real Time System software following amplification. All reactions exhibited a single distinct peak, confirming amplification specificity and the absence of non-specific products or primer-dimers. The representative curve shown corresponds to the reference gene β-actin.
