# Supplementary material for: Pyridoxine requirement of Pacific white shrimp (Penaeus vannamei) fed soybean meal based diet
Source: PLoS One. 2026 Jun 17;21(6):e0351680. doi: 10.1371/journal.pone.0351680 (PMC13274893; doi:10.1371/journal.pone.0351680)
Supplement: Attachment — Submitted filename: [Response Letter_Editor] PONE-D-26-08918.docx [file pone.0351680.s003.docx]

**Response Letter – ID: PONE-D-26-08918**

Pyridoxine requirement of Pacific white shrimp (*Penaeus vannamei*) fed soybean meal based diet

**Dear Editor In-Chief,**

Thank you for the thoughtful suggestions and comments on our manuscript. The authors have carefully revised the manuscript according to the comments/suggestions from reviewers and provided point-by-point responses below. Please note that all line numbers cited in the responses refer to the clean version of the revised manuscript. I anticipate your positive response and hope that you can find our manuscript suitable for publication in *PLOS ONE*.

Sincerely yours,

Kyeong-Jun Lee, Ph.D.

**Responses to the comments of Editor**

**1.  We note that you have provided funding information that is not currently declared in your Funding Statement. However, funding information should not appear in the Acknowledgments section or other areas of your manuscript. We will only publish funding information present in the Funding Statement section of the online submission form.**

**Answer:** We mistakenly included funding information in the Acknowledgments section. The funding information has been moved to the Funding Statement section and declared in the online submission form as requested.

**2. We note that your Data Availability Statement is currently as follows: All relevant data are within the manuscript and its Supporting Information files. Please confirm at this time whether or not your submission contains all raw data required to replicate the results of your study.**

**Answer:** We confirm that all data required to replicate the findings of this study are presented within the manuscript, including all numerical values, means, and standard deviations reported in the tables and figures. No additional data repository is required.

**3. In the online submission form, you indicated that; Data will be available on request. All PLOS journals now require all data underlying the findings described in their manuscript to be freely available to other researchers.**

**Answer:** We apologize for the incorrect data availability statement. All data underlying the findings of this study are presented within the manuscript itself, including all values reported in the tables and figures. The Data Availability Statement has been updated accordingly.

**4. Your ethics statement should only appear in the Methods section of your manuscript. If your ethics statement is written in any section besides the Methods, please move it to the Methods section and delete it from any other section. Please ensure that your ethics statement is included in your manuscript, as the ethics statement entered into the online submission form will not be published alongside your manuscript.**

**Answer:** We thank the editorial for this comment. The ethics statement has been moved to the Methods section of the revised manuscript. **(Lines 89-92)**
