# Supplementary material for: Pyridoxine requirement of Pacific white shrimp (Penaeus vannamei) fed soybean meal based diet
Source: PLoS One. 2026 Jun 17;21(6):e0351680. doi: 10.1371/journal.pone.0351680 (PMC13274893; doi:10.1371/journal.pone.0351680)
Supplement: Peer review history

# Peer review: Decision Letter 0

**DOI:** 10.1371/journal.pone.0351680.r001

**Authors:** Amel El Asely

12 Apr 2026

PONE-D-26-08918

Pyridoxine requirement of Pacific white shrimp (Penaeus vannamei) fed soybean meal based diet

PLOS One

Dear Dr. Lee,

Thank you for submitting your manuscript to PLOS ONE. After careful consideration, we feel that it has merit but does not fully meet PLOS ONE’s publication criteria as it currently stands. Therefore, we invite you to submit a revised version of the manuscript that addresses the points raised during the review process.

Please submit your revised manuscript by May 27 2026 11:59PM. If you will need more time than this to complete your revisions, please reply to this message or contact the journal office at plosone@plos.org. When you're ready to submit your revision, log on to https://www.editorialmanager.com/pone/ and select the 'Submissions Needing Revision' folder to locate your manuscript file.

Please include the following items when submitting your revised manuscript:

- A letter that responds to each point raised by the academic editor and reviewer(s). You should upload this letter as a separate file labeled 'Response to Reviewers'.
- A marked-up copy of your manuscript that highlights changes made to the original version. You should upload this as a separate file labeled 'Revised Manuscript with Track Changes'.
- An unmarked version of your revised paper without tracked changes. You should upload this as a separate file labeled 'Manuscript'.

If you would like to make changes to your financial disclosure, please include your updated statement in your cover letter. Guidelines for resubmitting your figure files are available below the reviewer comments at the end of this letter.

If applicable, we recommend that you deposit your laboratory protocols in protocols.io to enhance the reproducibility of your results. Protocols.io assigns your protocol its own identifier (DOI) so that it can be cited independently in the future. For instructions see: https://journals.plos.org/plosone/s/submission-guidelines#loc-laboratory-protocols. Additionally, PLOS ONE offers an option for publishing peer-reviewed Lab Protocol articles, which describe protocols hosted on protocols.io. Read more information on sharing protocols at https://plos.org/protocols?utm_medium=editorial-email&utm_source=authorletters&utm_campaign=protocols.

As the corresponding author, your ORCID iD is verified in the submission system and will appear in the published article. PLOS supports the use of ORCID, and we encourage all coauthors to register for an ORCID iD and use it as well. Please encourage your coauthors to verify their ORCID iD within the submission system before final acceptance, as unverified ORCID iDs will not appear in the published article. *Only*  the individual author can complete the verification step; PLOS staff *cannot*  verify ORCID iDs on behalf of authors.

We look forward to receiving your revised manuscript.

Kind regards,

Amel Mohamed El Asely

Academic Editor

PLOS One

**Journal Requirements:**

When submitting your revision, we need you to address these additional requirements.

1. Please ensure that your manuscript meets PLOS ONE's style requirements, including those for file naming. The PLOS ONE style templates can be found at

https://journals.plos.org/plosone/s/file?id=wjVg/PLOSOne_formatting_sample_main_body.pdf and

https://journals.plos.org/plosone/s/file?id=ba62/PLOSOne_formatting_sample_title_authors_affiliations.pdf

2. Thank you for stating the following in the Acknowledgments Section of your manuscript:

“This research was supported by the National Research Foundation of Korea (NRF) grand by the Korea government (2021R1A2C2008384) and Basic Science Research Program through the NRF funded by the Ministry of Education (RS-2019-NR040078).”

We note that you have provided funding information that is not currently declared in your Funding Statement. However, funding information should not appear in the Acknowledgments section or other areas of your manuscript. We will only publish funding information present in the Funding Statement section of the online submission form.

Please remove any funding-related text from the manuscript and let us know how you would like to update your Funding Statement. Currently, your Funding Statement reads as follows:

“The author(s) received no specific funding for this work.”

Please include your amended statements within your cover letter; we will change the online submission.

3. We note that your Data Availability Statement is currently as follows:

“All relevant data are within the manuscript and its Supporting Information files.”

Please confirm at this time whether or not your submission contains all raw data required to replicate the results of your study. Authors must share the “minimal data set” for their submission. PLOS defines the minimal data set to consist of the data required to replicate all study findings reported in the article, as well as related metadata and methods (https://journals.plos.org/plosone/s/data-availability#loc-minimal-data-set-definition).

For example, authors should submit the following data:

- The values behind the means, standard deviations and other measures reported;

- The values used to build graphs;

- The points extracted from images for analysis.

Authors do not need to submit their entire data set if only a portion of the data was used in the reported study.

If your submission does not contain these data, please either upload them as Supporting Information files or deposit them to a stable, public repository and provide us with the relevant URLs, DOIs, or accession numbers. For a list of recommended repositories, please see https://journals.plos.org/plosone/s/recommended-repositories.

If there are ethical or legal restrictions on sharing a de-identified data set, please explain them in detail (e.g., data contain potentially sensitive information, data are owned by a third-party organization, etc.) and who has imposed them (e.g., an ethics committee). Please also provide contact information for a data access committee, ethics committee, or other institutional body to which data requests may be sent. If data are owned by a third party, please indicate how others may request data access.

4. Your ethics statement should only appear in the Methods section of your manuscript. If your ethics statement is written in any section besides the Methods, please move it to the Methods section and delete it from any other section. Please ensure that your ethics statement is included in your manuscript, as the ethics statement entered into the online submission form will not be published alongside your manuscript.

5. If the reviewer comments include a recommendation to cite specific previously published works, please review and evaluate these publications to determine whether they are relevant and should be cited. There is no requirement to cite these works unless the editor has indicated otherwise.

[Note: HTML markup is below. Please do not edit.]

Reviewers' comments:

Reviewer's Responses to Questions

**Comments to the Author**

1. Is the manuscript technically sound, and do the data support the conclusions?

The manuscript must describe a technically sound piece of scientific research with data that supports the conclusions. Experiments must have been conducted rigorously, with appropriate controls, replication, and sample sizes. The conclusions must be drawn appropriately based on the data presented.

Reviewer #1: Yes

Reviewer #2: Yes

Reviewer #3: Partly

**********

2. Has the statistical analysis been performed appropriately and rigorously?

Reviewer #1: Yes

Reviewer #2: Yes

Reviewer #3: No

**********

3. Have the authors made all data underlying the findings in their manuscript fully available?

The PLOS Data policy requires authors to make all data underlying the findings described in their manuscript fully available without restriction, with rare exception (please refer to the Data Availability Statement in the manuscript PDF file). The data should be provided as part of the manuscript or its supporting information, or deposited to a public repository. For example, in addition to summary statistics, the data points behind means, medians and variance measures should be available. If there are restrictions on publicly sharing data—e.g. participant privacy or use of data from a third party—those must be specified.

Reviewer #1: Yes

Reviewer #2: Yes

Reviewer #3: No

**********

4. Is the manuscript presented in an intelligible fashion and written in standard English?

PLOS ONE does not copyedit accepted manuscripts, so the language in submitted articles must be clear, correct, and unambiguous. Any typographical or grammatical errors should be corrected at revision, so please note any specific errors here.

Reviewer #1: Yes

Reviewer #2: Yes

Reviewer #3: Yes

**********

5. Review Comments to the Author

Please use the space provided to explain your answers to the questions above. You may also include additional comments for the author, including concerns about dual publication, research ethics, or publication ethics. (Please upload your review as an attachment if it exceeds 20,000 characters)

Reviewer #1: There are some comments and necessary points must be met before publishing for paper improvements:

-Some editing for English language is required throughout the manuscript

mention the kit's Catalog number and reference used for

non-specific immune response and antioxidant capacity and Digestive enzyme analysis , methods should be in more detail.

Reviewer #2: - Please provide references for the selected dose of tetracycline hydrochloride used in this study.

- Please verify the sequence of the forward primer for the beta-actin gene listed in the table 2.

- Authors, could you explain the difference in growth performance as presented in Table 3, specifically between P150 and P75?

- Please indicate the size of the amplicons produced by each primer set for the various genes in Table 2.

- Please clarify how non-specific amplification of genes was ruled out in this study.

Reviewer #3: The manuscript has several ambiguities that need to be addressed before consideration. These are listed below;

1. The experimental design lacks adequate justification. The rationale behind the selection of treatments, such as the use of tetracycline as a control and the inclusion levels of pyridoxine, is not clearly explained. Without a strong scientific basis for these choices, it is difficult to assess whether the design appropriately addresses the research objectives.

2. The study does not clearly define a specific hypothesis or research question. The objectives appear descriptive rather than hypothesis-driven, which weakens the work's scientific direction and limits its contribution to theory- or mechanism-based understanding.

3. There is an insufficient mechanistic explanation of the results. While improvements in growth, antioxidant activity, or immunity are reported, the manuscript does not adequately explain the biological pathways through which pyridoxine exerts these effects. This results in a largely observational study rather than a mechanistic one.

4. The statistical analysis is inadequately described and potentially insufficient. Although methods such as ANOVA and regression are mentioned, there is no clear description of the statistical model, assumptions tested, or post hoc comparisons performed. This raises concerns about the robustness and reproducibility of the results.

5. There is limited validation of results across multiple parameters. The study relies heavily on biochemical and growth indicators but lacks complementary validation (e.g., molecular markers, gene expression, or histological evidence) to support its conclusions.

6. The sample size and experimental replication are not clearly justified. Without information on statistical power or replication strategy, it is difficult to determine whether the study is adequately powered to detect meaningful differences.

7. The discussion lacks critical comparison with existing literature. Although some references are cited, the manuscript does not adequately assess whether the findings align with or conflict with previous studies, nor does it explain any discrepancies.

8. There is a possible overinterpretation of results. The conclusions extend beyond the data presented, particularly in their suggestion of broad applications of pyridoxine, without sufficient supporting evidence or acknowledgment of study limitations.

9. The study does not adequately address potential confounding factors. Variables such as environmental conditions, feed composition variability, or biological variability among experimental units are not discussed, which may influence the results.

10. Also, the manuscript lacks a clear discussion of study limitations and external validity. There is no consideration of how the findings translate to real-world conditions or other species/systems, limiting the applicability of the research.

**********

6. PLOS authors have the option to publish the peer review history of their article (what does this mean?). If published, this will include your full peer review and any attached files.

If you choose “no”, your identity will remain anonymous but your review may still be made public.

**Do you want your identity to be public for this peer review?**  For information about this choice, including consent withdrawal, please see our Privacy Policy.

Reviewer #1: No

Reviewer #2: **Yes:**  Khawaja Ashfaque Ahmed

Reviewer #3: No

**********

[NOTE: If reviewer comments were submitted as an attachment file, they will be attached to this email and accessible via the submission site. Please log into your account, locate the manuscript record, and check for the action link "View Attachments". If this link does not appear, there are no attachment files.]

To ensure your figures meet our technical requirements, please review our figure guidelines: https://journals.plos.org/plosone/s/figures

You may also use PLOS’s free figure tool, NAAS, to help you prepare publication quality figures: https://journals.plos.org/plosone/s/figures#loc-tools-for-figure-preparation.

NAAS will assess whether your figures meet our technical requirements by comparing each figure against our figure specifications.

# Author comment: Author response to Decision Letter 1

**DOI:** 10.1371/journal.pone.0351680.r002

12 May 2026

Response Letter – ID: PONE-D-26-08918

Pyridoxine requirement of Pacific white shrimp (Penaeus vannamei) fed soybean meal based diet

Dear Editor In-Chief,

Thank you for the thoughtful suggestions and comments on our manuscript. The authors have carefully revised the manuscript according to the comments/suggestions from reviewers and provided point-by-point responses below. Please note that all line numbers cited in the responses refer to the clean version of the revised manuscript. I anticipate your positive response and hope that you can find our manuscript suitable for publication in PLOS ONE.

Sincerely yours,

Kyeong-Jun Lee, Ph.D.

Responses to the comments of Reviewer #1

1. Some editing for English language is required throughout the manuscript

Answer: We thank the reviewer for this comment. The manuscript has been carefully revised to improve English language and clarity throughout.

2. Mention the kit's Catalog number and reference used for non-specific immune response and antioxidant capacity and Digestive enzyme analysis, methods should be in more detail.

Answer: Thank you for this valuable comment. We have revised the manuscript to include the catalog numbers and references for all commercial assay kits used for non-specific immune response, antioxidant capacity, and digestive enzyme analyses. In addition, more detailed descriptions of each assay procedure have been provided to improve clarity and reproducibility. These revisions are reflected in the Materials and Methods section of the revised manuscript (Lines 178-234).

Responses to the comments of Reviewer #2.

1. Please provide references for the selected dose of tetracycline hydrochloride used in this study.

Answer: We thank the reviewer for this valuable comment. References supporting the selected tetracycline hydrochloride level have been added to the revised manuscript. Previous studies have shown that tetracycline-class antibiotics, such as oxytetracycline, can be used in P. vannamei at dietary levels up to 0.45% without adverse effects on growth or health (Bray et al., 2006), and can modulate intestinal microbiota. In addition, tetracycline hydrochloride at 0.3% has been reported to have no negative effects on growth or survival in P. vannamei (Medagoda and Lee, 2025). Therefore, the inclusion level used in this study (0.4%) falls within the previously reported safe range and was considered appropriate. This has been added in Lines 94-97.

2. Please verify the sequence of the forward primer for the beta-actin gene listed in the table 2.

Answer: Thank you for this valuable comment. We rechecked the forward primer sequence of the β-actin gene and confirmed that an inadvertent typographical error was present in Table 2. The extra letter “F” was mistakenly included at the beginning of the forward primer sequence. This has now been corrected in the revised manuscript.

3. Authors, could you explain the difference in growth performance as presented in Table 3, specifically between P150 and P75?

Answer: We thank the reviewer for this comment. As observed in some fish species, growth performance in the present study increased with increasing dietary pyridoxine levels up to an optimal point, followed by a decline at higher inclusion levels. Although the underlying mechanisms in shrimp remain unclear, the decreased growth at excessive pyridoxine levels may be associated with metabolic imbalance, as high concentrations of pyridoxine can interfere with its conversion to the active form, pyridoxal-5'-phosphate, potentially reducing metabolic efficiency. This explanation has been added to the Discussion section of the revised manuscript. (Lines 503-512)

4. Please indicate the size of the amplicons produced by each primer set for the various genes in Table 2.

Answer: We thank the reviewer for this comment. The amplicon sizes for all primer sets have been added to Table 2 in the revised manuscript.

5. Please clarify how non-specific amplification of genes was ruled out in this study.

Answer: We appreciate the reviewer’s comment. Amplification specificity was evaluated using melt curve (dissociation curve) analysis following qPCR. All reactions exhibited a single sharp peak without any secondary peaks, indicating the absence of non-specific amplification or primer-dimer formation. A representative melt curve (β-actin) has been provided as Supplementary Fig. S1 to support this observation. The corresponding description has been added to the revised manuscript (Lines 246-248).

Responses to the comments of Reviewer #3.

1. The experimental design lacks adequate justification. The rationale behind the selection of treatments, such as the use of tetracycline as a control and the inclusion levels of pyridoxine, is not clearly explained. Without a strong scientific basis for these choices, it is difficult to assess whether the design appropriately addresses the research objectives.

Answer: We thank you for this valuable comment. We have revised the manuscript to clarify the rationale behind the experimental design, including the selection of control groups, tetracycline application, and pyridoxine inclusion levels.

The control diet (Con) without pyridoxine supplementation was used as the basal control to evaluate the baseline level of pyridoxine supplied from feed ingredients. In contrast, the tetracycline-supplemented group (Con−) was not used as a conventional control, but rather as a functional control to suppress intestinal microbiota and to assess the contribution of microbial pyridoxine synthesis. This approach has been adopted in previous studies investigating vitamin B12 requirements in P. vannamei shrimp where antibiotics were used to distinguish between dietary and microbially derived vitamin sources (Medagoda and Lee, 2025).

The inclusion level of tetracycline hydrochloride (0.4%) was selected based on previous studies. Oxytetracycline has been reported to be safely applied up to 0.45% in shrimp without adverse effects on growth performance, survival, or hepatopancreatic condition (Bray et al., 2006), and tetracycline hydrochloride has been used at 0.3% without negative effects (Medagoda and Lee, 2025). These findings support that the level used in the present study falls within a safe and effective range for modulating intestinal microbiota. This has been added in Lines 74-79, 94-97.

The graded dietary pyridoxine levels (0–150 mg/kg) were established based on previously reported requirement ranges for P. vannamei, which have been suggested to be approximately 110–150 mg/kg diet (Li et al., 2010; Cui et al., 2016). The range of 0–150 mg/kg was designed to encompass both deficient and supraoptimal levels, enabling accurate estimation of the dietary requirement through dose-response analysis. Additional clarification regarding the selection of dietary pyridoxine levels has been added in Lines 99-103.

2. The study does not clearly define a specific hypothesis or research question. The objectives appear descriptive rather than hypothesis-driven, which weakens the work's scientific direction and limits its contribution to theory- or mechanism-based understanding.

Answer: Thank you for this insightful comment. The hypothesis of this study was originally included in the manuscript; however, we agree that it was not stated with sufficient clarity. Accordingly, we have revised the Introduction to present the hypothesis more explicitly. Specifically, we hypothesized that the pyridoxine requirement of P. vannamei would differ when shrimp are fed a soybean meal-based diet compared to a fish meal-based formulation, due to differences in nutrient composition and potential changes in metabolic utilization and microbial contribution to vitamin B₆ synthesis. (Lines 79-82).

3. There is an insufficient mechanistic explanation of the results. While improvements in growth, antioxidant activity, or immunity are reported, the manuscript does not adequately explain the biological pathways through which pyridoxine exerts these effects. This results in a largely observational study rather than a mechanistic one.

Answer: Thank you for this valuable comment. Accordingly, we have revised the Discussion to incorporate more detailed mechanistic interpretations, including the role of PLP-dependent enzymes in amino acid metabolism and protein turnover (growth), the transsulfuration pathway linking CBS and CGL upregulation to glutathione synthesis (antioxidant capacity), and pyridoxine's involvement in one-carbon metabolism and immune-related signaling pathways (innate immunity). These revisions have been incorporated throughout the Discussion section of the revised manuscript.

4. The statistical analysis is inadequately described and potentially insufficient. Although methods such as ANOVA and regression are mentioned, there is no clear description of the statistical model, assumptions tested, or post hoc comparisons performed. This raises concerns about the robustness and reproducibility of the results.

Answer: We thank the reviewer for this comment. The Statistical Analysis section has been revised to include a more detailed description, including the experimental design, experimental unit, assumption tests (Shapiro-Wilk test for normality and Levene's test for homogeneity of variances), arcsine transformation for percentage data, post hoc comparisons, and additional statistical approaches. These revisions have been incorporated in the Statistical Analysis section of the revised manuscript. (Lines 270-274).

5. There is limited validation of results across multiple parameters. The study relies heavily on biochemical and growth indicators but lacks complementary validation (e.g., molecular markers, gene expression, or histological evidence) to support its conclusions

Answer: Thank you for this valuable comment. We would like to clarify that this study includes multiple complementary approaches beyond growth and biochemical indicators, including gene expression analysis and histological evaluation, which provide molecular- and tissue-level validation of the results. The Discussion has been revised to better link biochemical, molecular, and histological findings for a more comprehensive interpretation of the revised manuscript.

6. The sample size and experimental replication are not clearly justified. Without information on statistical power or replication strategy, it is difficult to determine whether the study is adequately powered to detect meaningful differences.

Answer: We thank the reviewer for this comment. We have clarified the experimental replication and statistical strategy in the revised manuscript. Each treatment was conducted in quadruplicate tanks, and each tank was considered as the experimental unit for statistical analysis. Measurements from multiple shrimp within each tank were averaged to represent one replicate. This information has been added to the Statistical Analysis section (Lines 268–270). In addition, the number of shrimp sampled per tank for each analysis has been specified in the Sample Collection section, and the replication of experimental units is also indicated in the table footnotes.

7. The discussion lacks critical comparison with existing literature. Although some references are cited, the manuscript does not adequately assess whether the findings align with or conflict with previous studies, nor does it explain any discrepancies

Answer: Thank you for this valuable comment. Discussion has been revised to more clearly highlight the consistency and discrepancies between the present results and previous findings, along with possible explanations for these differences.

8. There is a possible overinterpretation of results. The conclusions extend beyond the data presented, particularly in their suggestion of broad applications of pyridoxine, without sufficient supporting evidence or acknowledgment of study limitations.

Answer: Thank you for this comment. The Conclusion has been revised to avoid overgeneralization and to better reflect the scope of the present study. In addition, a limitations paragraph has been added to the Discussion acknowledging that the applicability of the results may be limited by environmental and management factors, that findings are based on a single species and specific diet formulation, and that further validation under commercial-scale conditions is warranted (Lines 650-655, 666-667).

9. The study does not adequately address potential confounding factors. Variables such as environmental conditions, feed composition variability, or biological variability among experimental units are not discussed, which may influence the results.

Answer: Thank you for your comment. Potential confounding factors were carefully controlled in this study. Shrimp were obtained from the same local hatchery and similar-sized individuals were selected to minimize biological variability. All tanks were maintained under identical rearing conditions throughout the feeding trial, and water quality parameters were consistently monitored. All experimental diets were formulated to be identical except for pyridoxine levels. These details are described in the Materials and Methods (Feeding trial) section of the manuscript.

10. Also, the manuscript lacks a clear discussion of study limitations and external validity. There is no consideration of how the findings translate to real-world conditions or other species/systems, limiting the applicability of the research.

Answer: Thanks for your comment. A limitations paragraph has been added to the Discussion acknowledging that the applicability of the results may be limited by environmental and management factors, that the findings are based on a single species and specific diet formulation, and that further validation under commercial-scale farming conditions is warranted. These revisions have been incorporated in the revised manuscript. (Lines 650-655).

Attachment Submitted filename: [Response Letter_Editor] PONE-D-26-08918.docx

# Peer review: Decision Letter 1

**DOI:** 10.1371/journal.pone.0351680.r003

**Authors:** Amel El Asely

31 May 2026

Pyridoxine requirement of Pacific white shrimp (Penaeus vannamei) fed soybean meal based diet

PONE-D-26-08918R1

Dear Dr. Kyeong-Jun Lee,

We’re pleased to inform you that your manuscript has been judged scientifically suitable for publication and will be formally accepted for publication once it meets all outstanding technical requirements.

Within one week, you’ll receive an e-mail detailing the required amendments. When these have been addressed, you’ll receive a formal acceptance letter and your manuscript will be scheduled for publication.

An invoice will be generated when your article is formally accepted. Please note, if your institution has a publishing partnership with PLOS and your article meets the relevant criteria, all or part of your publication costs will be covered. Please make sure your user information is up-to-date by logging into Editorial Manager at Editorial Manager® and clicking the ‘Update My Information' link at the top of the page. For questions related to billing, please contact billing support.

If your institution or institutions have a press office, please notify them about your upcoming paper to help maximize its impact. If they’ll be preparing press materials, please inform our press team as soon as possible -- no later than 48 hours after receiving the formal acceptance. Your manuscript will remain under strict press embargo until 2 pm Eastern Time on the date of publication. For more information, please contact onepress@plos.org.

Kind regards,

Amel Mohamed El Asely

Academic Editor

PLOS One

Additional Editor Comments (optional):

Reviewers' comments:

Reviewer's Responses to Questions

**Comments to the Author**

1. If the authors have adequately addressed your comments raised in a previous round of review and you feel that this manuscript is now acceptable for publication, you may indicate that here to bypass the “Comments to the Author” section, enter your conflict of interest statement in the “Confidential to Editor” section, and submit your "Accept" recommendation.

Reviewer #1: All comments have been addressed

Reviewer #2: All comments have been addressed

Reviewer #3: All comments have been addressed

**********

2. Is the manuscript technically sound, and do the data support the conclusions?

The manuscript must describe a technically sound piece of scientific research with data that supports the conclusions. Experiments must have been conducted rigorously, with appropriate controls, replication, and sample sizes. The conclusions must be drawn appropriately based on the data presented.

Reviewer #1: Yes

Reviewer #2: Yes

Reviewer #3: Yes

**********

3. Has the statistical analysis been performed appropriately and rigorously?

Reviewer #1: Yes

Reviewer #2: Yes

Reviewer #3: Yes

**********

4. Have the authors made all data underlying the findings in their manuscript fully available?

The PLOS Data policy requires authors to make all data underlying the findings described in their manuscript fully available without restriction, with rare exception (please refer to the Data Availability Statement in the manuscript PDF file). The data should be provided as part of the manuscript or its supporting information, or deposited to a public repository. For example, in addition to summary statistics, the data points behind means, medians and variance measures should be available. If there are restrictions on publicly sharing data—e.g. participant privacy or use of data from a third party—those must be specified.

Reviewer #1: Yes

Reviewer #2: Yes

Reviewer #3: Yes

**********

5. Is the manuscript presented in an intelligible fashion and written in standard English?

PLOS ONE does not copyedit accepted manuscripts, so the language in submitted articles must be clear, correct, and unambiguous. Any typographical or grammatical errors should be corrected at revision, so please note any specific errors here.

Reviewer #1: Yes

Reviewer #2: Yes

Reviewer #3: Yes

**********

6. Review Comments to the Author

Please use the space provided to explain your answers to the questions above. You may also include additional comments for the author, including concerns about dual publication, research ethics, or publication ethics. (Please upload your review as an attachment if it exceeds 20,000 characters)

Reviewer #1: the authors have adequately addressed the comments raised in a previous round of review and I feel that this manuscript is now acceptable for publication

the manuscript is technically sound, and the data support the conclusions

It's a nice job to revise the manuscript and address the comments.

Reviewer #2: (No Response)

Reviewer #3: Dear Author(s),

I have carefully evaluated the revised manuscript and the authors’ detailed responses to the reviewers’ comments. The authors have adequately addressed the concerns raised during the review process and have made substantial improvements to the manuscript. The revised manuscript is now scientifically sound and well-organized, and the methodology, statistical analyses, discussion, and interpretation of results have been significantly strengthened. The additional clarifications regarding experimental design, replication strategy, study limitations, and comparison with previous literature have improved the overall quality and clarity of the work. In my opinion, the manuscript is suitable for publication in its present form, and I therefore recommend acceptance. I deeply appreciate the authors’ timely responses to all the comments and suggestions raised by the reviewers.

**********

7. PLOS authors have the option to publish the peer review history of their article (what does this mean?). If published, this will include your full peer review and any attached files.

If you choose “no”, your identity will remain anonymous but your review may still be made public.

**Do you want your identity to be public for this peer review?**  For information about this choice, including consent withdrawal, please see our Privacy Policy.

Reviewer #1: No

Reviewer #2: **Yes:**  Khawaja Ashfaque Ahmed

Reviewer #3: No

**********

# Editor report: Acceptance letter

**DOI:** 10.1371/journal.pone.0351680.r004

**Authors:** Amel El Asely

PONE-D-26-08918R1

PLOS One

Dear Dr. Lee,

I'm pleased to inform you that your manuscript has been deemed suitable for publication in PLOS One. Congratulations! Your manuscript is now being handed over to our production team.

At this stage, our production department will prepare your paper for publication. This includes ensuring the following:

* All references, tables, and figures are properly cited

* All relevant supporting information is included in the manuscript submission,

* There are no issues that prevent the paper from being properly typeset

You will receive further instructions from the production team, including instructions on how to review your proof when it is ready. Please keep in mind that we are working through a large volume of accepted articles, so please give us a few days to review your paper and let you know the next and final steps.

Lastly, if your institution or institutions have a press office, please let them know about your upcoming paper now to help maximize its impact. If they'll be preparing press materials, please inform our press team within the next 48 hours. Your manuscript will remain under strict press embargo until 2 pm Eastern Time on the date of publication. For more information, please contact onepress@plos.org.

You will receive an invoice from PLOS for your publication fee after your manuscript has reached the completed accept phase. If you receive an email requesting payment before acceptance or for any other service, this may be a phishing scheme. Learn how to identify phishing emails and protect your accounts at https://explore.plos.org/phishing.

If we can help with anything else, please email us at customercare@plos.org.

Thank you for submitting your work to PLOS ONE and supporting open access.

Kind regards,

PLOS ONE Editorial Office Staff

on behalf of

Prof. Amel Mohamed El Asely

Academic Editor

PLOS One
